# Supplementary material for: DUSP16 promotes cancer chemoresistance through regulation of mitochondria-mediated cell death
Source: Nat Commun. 2021 Apr 16;12:2284. doi: 10.1038/s41467-021-22638-7 (PMC8052345; doi:10.1038/s41467-021-22638-7)
Supplement: Supplementary file 1 — Supplementary Information [file 41467_2021_22638_MOESM1_ESM.pdf]

Supplementary Information

Supplementary Table 1. Baseline characteristics of the head and neck squamous cell carcinoma (HNSCC) population, classified according to DUSP16 scoring.

**Characteristic**	**Low DUSP16 (<150) (n=19)**	**High DUSP16 (≥150) (n=31)**
Age	55 (25 to 86)	55 (29 to 74)
Gender		
Male	14	22
Female	5	9
Stage (%)		
2	1 (5)	2 (6)
3	4 (21)	1 (3)
4	13 (68)	27 (87)
unknown	1(5)	1(3)
Site of disease (%)		
Larynx	5 (26)	9 (29)
Hypopharynx	1 (5)	3 (10)
Oropharynx	3 (16)	5 (16)
Oral cavity	9 (47)	12 (39)
Nasal cavity	1 (5)	2 (6)
HPV status (%)		
p16 positive	4 (22%)	6 (19%)

Supplementary Table 2. List of primers used in this study and their sequences

**Gene**	**Sequences**	**Purpose**
DUSP16	Forward: 5’-AAAAAACTCGAGATGGCCCATGAGATGATTGGAACTCAAATTGTTACTG-3’	For cloning, with an XhoI site.
	Reverse: 5’-AAAAAAGCGGCCGCTCAGGAGACCTCAATGATTTCCATG-3’	With a NotI site.
BAX sgRNA	Forward: 5’-CACCGGTTTCATCCAGGATCGAGCA-3’	For knockout.
	Reverse: 5’-AAACTGCTCGATCCTGGATGAAACC-3’	
ACTIN	Forward: 5’-CCCCGCGAGCACAGAG-3’	For qPCR.
	Reverse: 5’-ATCATCCATGGTGGTGAGCTGGC-3’	
DUSP16	Forward: 5’-GTTGACATTGATTGCAGT CAGAAG-3’	For qPCR.
	Reverse: 5’-CAAGCAGGTGAACAGAGTTGAAG-3’	

Supplementary Table 3. Primary antibody list for immunoblotting

Antibody	Dilution in 5% milk/1xTBST	Manufacturer and cat. number
β-actin	1:1000	Cell Signalling #4970
c-myc	1:1000	Abcam#32072
Cleaved Caspase 3	1:1000	Cell Signalling #9661
ERK	1:1000	Cell Signalling #4695
JNK	1:1000	Cell Signalling #4671
DUSP16	1:500	Abcam #ab15698
p38	1:1000	Cell Signalling #9212
Phospho-ERK	1:1000	Cell Signalling #4377
Phospho-JNK	1:1000	Cell Signalling #9252
Phospho-p38	1:1000	Cell Signalling #9215
Bax	1:1000	Cell Signalling #2772
VDAC	1:1000	Cell Signalling #4661
ATF-2	1:1000	Cell Signalling #9226

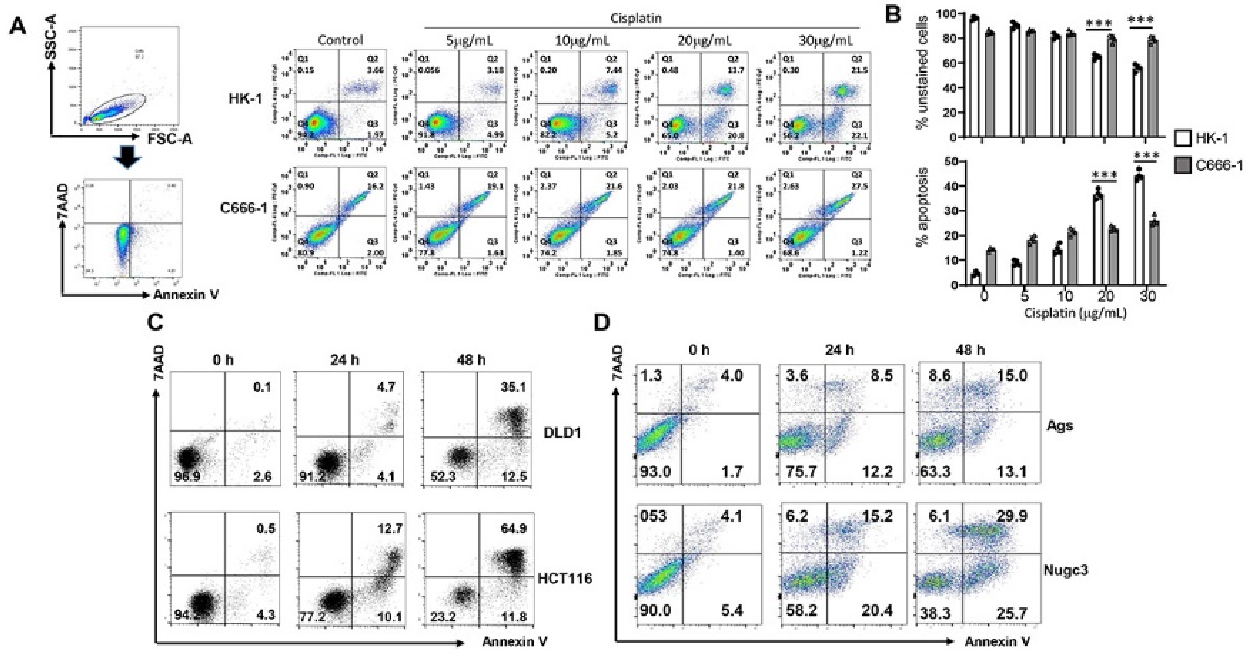

Supplementary Figure 1. DUSP16 protein expression level in NPC cells is associated with sensitivity to cisplatin. (**A**)The NPC cell lines HK-1 and C666-1 cells were treated with various concentrations of cisplatin for 24 hours. Total cell population was gated to live and apoptotic cells. Cell apoptosis was determined by Annexin V and 7ADD staining followed by flow cytometric analysis. (**B**) Bar charts show the percentage of apoptotic and surviving (unstained) cells with mean values ± SEM, n=3 biologically independent samples. The data is representative of 3 experiments with similar results. Statistical analysis was performed using two-tailed unpaired *t*-test. *** $P < 0.001$. (**C-D**) Colorectal DLD1 and HCT116 cells (**C**) and gastric Ags and Nugc3 cells (**D**) were treated with cisplatin for 24 and 48 hours to assess cell apoptosis by Annexin V and 7ADD staining followed by flow cytometric analysis. The data is representative of 3 experiments with similar results.

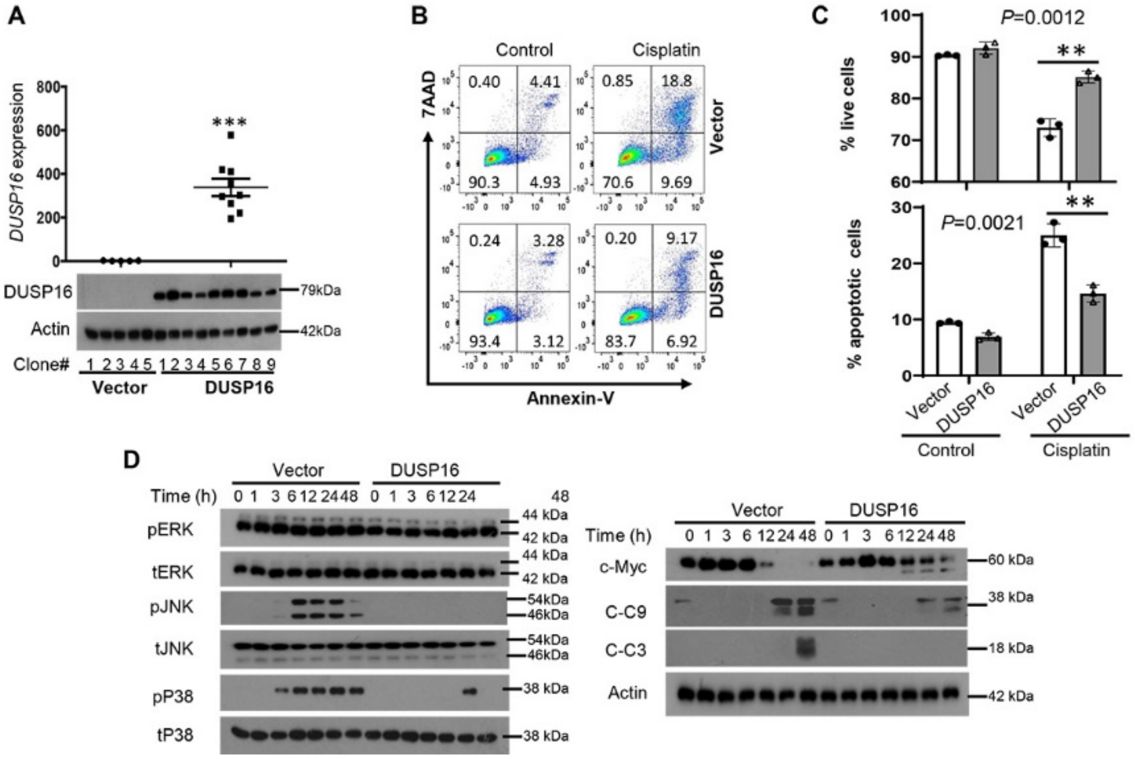

Supplementary Figure 2. *DUSP16* overexpression reduces apoptosis in MDA-MB-231 breast cancer cells in response to cisplatin. (**A**) mRNA and protein levels of DUSP16 in cells transfected with vector or *DUSP16* construct were quantified by qPCR and Western blot analysis respectively. Dot plots show significantly increased *DUSP16* expression in *DUSP16*-overexpression clones (n=9) compared to that in vector transfected clones (n=5). Statistical analysis was performed using two-tailed unpaired t-test. *** $P < 0.001$. (**B**) Cell apoptosis after cisplatin treatment was analyzed by Annexin V/7AAD staining and flow cytometry. (**C**) Percentages of surviving (unstained) and apoptotic cells (mean ± SEM, n=3 biologically independent samples) were quantified. Statistical analysis was performed using two-tailed unpaired t-test. (**D**) Expression of p-MAPKs, total MAPKs, c-Myc, cleaved caspase 9 (C-C9) and cleaved caspase 3 (C-C3) in vector- or *DUSP16*-overexpressing MDA-MB-231 cells was analyzed by Western blot analysis. The data is representative of 3 experiments with similar results. Source data are provided as a Source Data file.

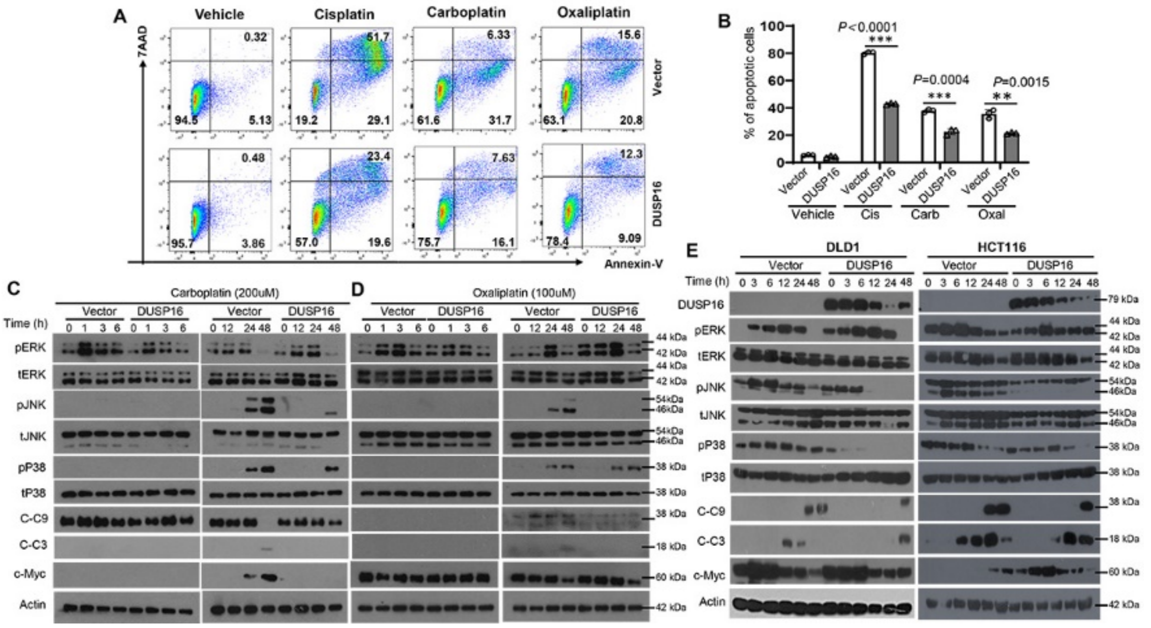

Supplementary Figure 3. ***DUSP16*** **overexpression leads to reduced apoptosis in response to cisplatin, carboplatin and oxalipatin treatments in various types of cancer cells.** (**A**) Comparison of apoptosis induction between vector- and DUSP16-transfected DLD-1 cells in response to cisplatin, carboplatin or oxaliplatin treatments by flow cytometry analysis. (**B**) Bar chart shows the percentage of apoptotic cells (mean ± SEM, n=3 biologically independent samples). Statistical analysis was performed using two-tailed unpaired *t*-test. $^{**}P < 0.001$. The data is representative of 3 experiments with similar results. (**C-D**) *DUSP16* overexpression suppresses intrinsic apoptotic pathway in HK-1 cells in response to carboplatin or oxaliplatin. Control or *DUSP16* overexressing HK-1 cells were treated with carboplatin (**C**) or oxaliplatin (**D**). Western blot analysis of p-MAPKs, total MAPKs, c-Myc, cleaved caspase 9 and cleaved caspase 3 were carried out. (**E**) DUSP16 dephosphorylates JNK and p38 to suppress intrinsic apoptotic pathway in DLD-1 and HCT116 cells in response to carboplatin. Control or DUSP16 overexpressing DLD-1 or HCT116 cells were treated with carboplatin. Levels of the p-MAPKs, total MAPKs, c-Myc, C-C9 and C-C3 were analyzed by immunoblotting. The data is representative of 2 experiments with similar results. Source data are provided as a Source Data file.

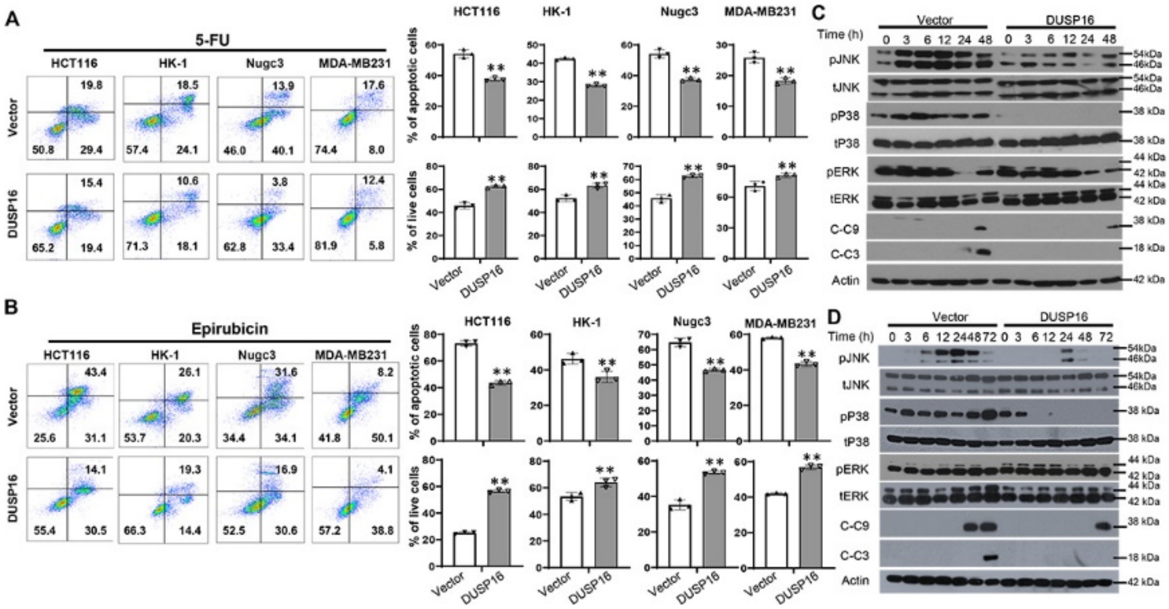

Supplementary Figure 4. Overexpression of *DUSP16* resulted in resistance to 5-fluorouracil and epirubicin in various cancer cells. (A-B) Vector- and *DUDP16*-expressing HCT116, HK-1, Nugc3 and MDA-MB231 cells were treated with 5-fluorouracial (5-FU) (**A**) or epirubicin (**B**) for 48 hours to determine cell apoptosis. Bar charts show the percentage of apoptotic and surviving cells (mean ± SEM, n=3 biologically independent samples). Statistical analysis was performed using two-tailed unpaired *t*-test. $**P<0.001$. The data is representative of 3 experiments with similar results. **(C-D)** Activation of JNK, p38 and ERK, and expression of C-C9 and C-C3 in HCT116 cells (C) and Nugc3 (D) were analyzed by western blot. The data is representative of 2 experiments with similar results. Source data are provided as a Source Data file.

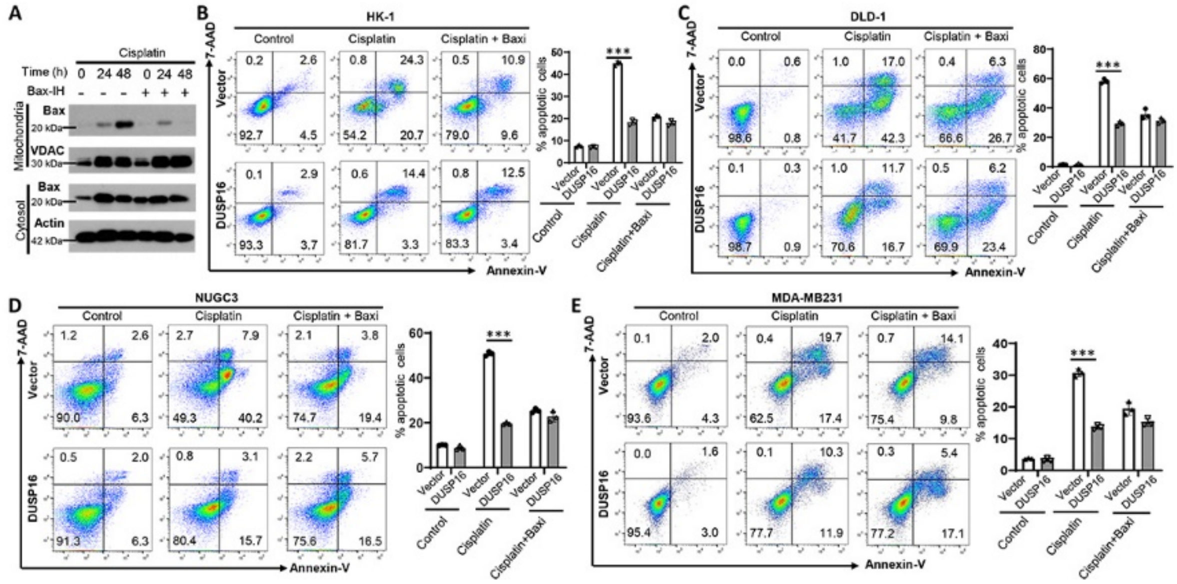

Supplementary Figure 5. BAX is essential for DUSP16-mediated cisplatin resistance in various types of cancer cells. **A**. HK-1 cells were treated with cisplatin with or without BAX inhibitor Peptide V5 (300µM). Cells were harvested at the indicated time points post-treatment to isolate mitochondria for examination of BAX accumulation in the mitochondria by western blot. The data is representative of 3 experiments with similar results. (**B-E**). Vector- and *DUSP16*-expressing NPC HK-1 cells (**B**), CRC DLD-1 cells (**C**), gastric cancer NUGC3 cells (**D**), and breast cancer MDA-MB231 cells (**E**) were treated with cisplatin with or without BAX inhibitor (Baxi) for 48 hours for examination of cell death by Annexin-V and 7-AAD staining followed by flow cytometry analysis. Bar charts on the right panels of (**B-C)** show the percentage of apoptotic cells (mean ± SEM, n=3 biologically independent samples). Bar charts on the right panel of (**D-E**) show the percentage of apoptotic cells of three samples in the representative experiment (mean ± SEM). Statistical analysis was performed using two-tailed unpaired *t*-test. The data is representative of 2 (**D-E**) and 3 (**A-C**) experiments with similar results. *** $P \leq 0.0001$. Source data are provided as a Source Data file.

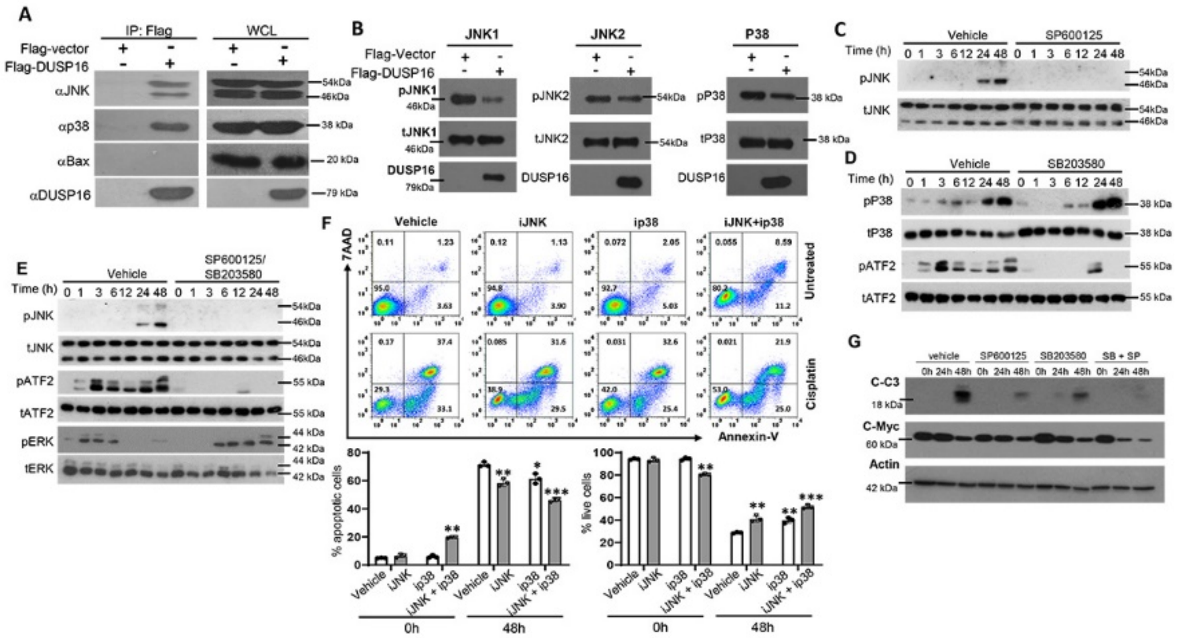

Supplementary Figure 6. DUSP16 regulates cancer cell apoptosis to cisplatin through JNK and p38. (**A**). HEK293T cells were transfected with either Flag-vector or Flag-*DUSP16* plasmids. One day post transfection, cells were harvested to prepare lysates. A portion of the cell lysate was used as whole cell lysate while the rest was used for pulldown with FLAG® tag Polyclonal Antibody, Magnetic beads (Pierce). The samples were then analyzed by western blot. (**B**) HEK293 T cells were transfected with Flag-tagged *JNK1*, JNK2 or *P38* (3 μg DNA each). Cells were stimulated with 100 ng/mL EGF for 10 min to induce phosphorylation of MAPKs before total protein was harvested. The Flag-tagged proteins were purified by immunoprecipitation using anti-Flag magnetic beads (Sigma). All the protein bound beads were aliquoted equally and used in the respective dephosphorylation reactions by incubation with recombinant DUSP16 protein in dephosphorylation buffer containing 50 mM HEPES, 100 mM NaCl, 1 mM $MnCl_2$, 2 mM DTT, 0.1 mM EGTA and 0.025% Tween 20 followed by incubation at 37 ºC for 2.5 hours. Phosphorylation of MAPKs was analyzed by western blot. (**C-F**) JNK and p38 inhibition suppresses apoptosis in HK-1 cells in response to cisplatin. HK-1 cells were treated with cisplatin for 48 hours, or pre-treated with JNK inhibitor SP600125 (iJNK) (**C**), p38 inhibitor SB203580 (ip38) (**D**), or both (**E**) for 1 hour followed by cisplatin

for 48 hours. Activation of JNK, p38, or both was assessed by Western blot analysis. Cell apoptosis was assessed by Annexin V/7-AAD staining and flow cytometry with or without cisplatin treatment (**F**). Bar charts show the percentage of apoptotic and surviving (unstained) cells of three samples in the representative experiment (mean ± SEM). Statistical analysis was performed using two-tailed unpaired *t*-test. *P<0.05, **P<0.001, ***P<0.0001 (mean ± SD). The data is representative of 2 experiments with similar results. (**G**) HK-1 cells were treated with cisplatin or pre-treated with the MAPK inhibitors followed by cisplatin for 24 or 48 hours. C-c3 and c-Myc expression were detected by Western blot analysis. The data is representative of 3 experiments with similar results. Source data are provided as a Source Data file.

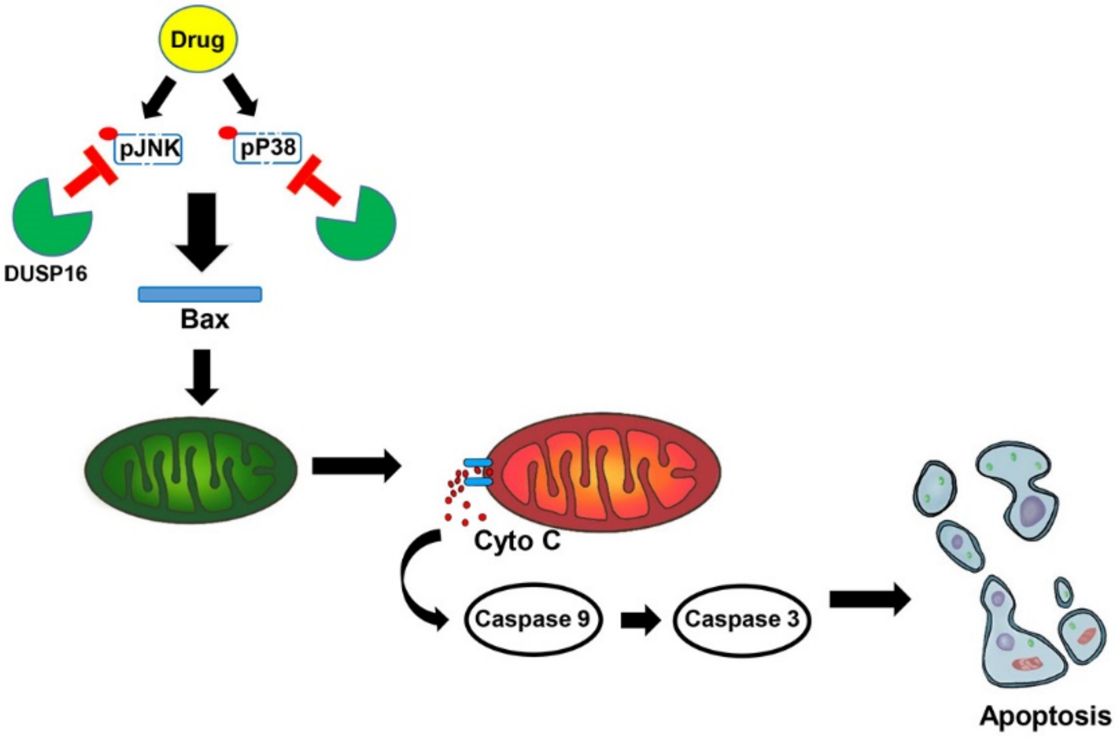

Supplementary Figure 7. **DUSP16 regulates p38/JNK-BAX signalling to mediate chemoresistance in cancer.** Chemotherapy drug treatment of cancer cells results in activation of JNK and p38 to promote BAX activation and translocation to mitochondria for apoptosis. The treatment also induces the expression of DUSP16 which dephosphorylates both JNK and p38 to suppress BAX activation and mitochondria-mediated apoptosis. High expression of DUSP16 could therefore lead to chemoresistance.
