# Supplementary material for: DUSP16 promotes cancer chemoresistance through regulation of mitochondria-mediated cell death
Source: Nat Commun. 2021 Apr 16;12:2284. doi: 10.1038/s41467-021-22638-7 (PMC8052345; doi:10.1038/s41467-021-22638-7)
Supplement: Supplementary file 3 — Reporting Summary [file 41467_2021_22638_MOESM3_ESM.pdf]

nature research

Corresponding author(s): Yongliang Zhang, Xiaohong Xu

Last updated by author(s): Feb 20, 2021

Reporting Summary

Nature Research wishes to improve the reproducibility of the work that we publish. This form provides structure for consistency and transparency in reporting. For further information on Nature Research policies, see Authors & Referees and the Editorial Policy Checklist .

Statistics

For all statistical analyses, confirm that the following items are present in the figure legend, table legend, main text, or Methods section.

n/a	Confirmed	
☐	☒	The exact sample size (*n*) for each experimental group/condition, given as a discrete number and unit of measurement
☐	☒	A statement on whether measurements were taken from distinct samples or whether the same sample was measured repeatedly
☐	☒	The statistical test(s) used AND whether they are one- or two-sided<br>*Only common tests should be described solely by name; describe more complex techniques in the Methods section.*
☒	☐	A description of all covariates tested
☒	☐	A description of any assumptions or corrections, such as tests of normality and adjustment for multiple comparisons
☐	☒	A full description of the statistical parameters including central tendency (e.g. means) or other basic estimates (e.g. regression coefficient) AND variation (e.g. standard deviation) or associated estimates of uncertainty (e.g. confidence intervals)
☐	☒	For null hypothesis testing, the test statistic (e.g. *F*, *t*, *r*) with confidence intervals, effect sizes, degrees of freedom and *P* value noted<br>*Give P values as exact values whenever suitable.*
☒	☐	For Bayesian analysis, information on the choice of priors and Markov chain Monte Carlo settings
☒	☐	For hierarchical and complex designs, identification of the appropriate level for tests and full reporting of outcomes
☒	☐	Estimates of effect sizes (e.g. Cohen's *d*, Pearson's *r*), indicating how they were calculated

Our web collection on statistics for biologists contains articles on many of the points above.

Software and code

Policy information about availability of computer code

Data collection	No software was used for data collection.
Data analysis	IBM SPSS Statistic 19.0 and GraphPad Prism software.

For manuscripts utilizing custom algorithms or software that are central to the research but not yet described in published literature, software must be made available to editors/reviewers. We strongly encourage code deposition in a community repository (e.g. GitHub). See the Nature Research guidelines for submitting code & software for further information.

Data

Policy information about availability of data

All manuscripts must include a data availability statement. This statement should provide the following information, where applicable:

- Accession codes, unique identifiers, or web links for publicly available datasets
- A list of figures that have associated raw data
- A description of any restrictions on data availability

The authors declare that the data supporting the findings of this study are available within the article and its supplementary information files.

Field-specific reporting

Please select the one below that is the best fit for your research. If you are not sure, read the appropriate sections before making your selection.

☒ Life sciences ☐ Behavioural & social sciences ☐ Ecological, evolutionary & environmental sciences

For a reference copy of the document with all sections, see nature.com/documents/nr-reporting-summary-flat.pdf

Life sciences study design

All studies must disclose on these points even when the disclosure is negative.

Sample size	Head and Neck squamous cell carcinoma (n=50) and breast cancer (n=113) sample sizes were determined upon availability of sample numbers.
Data exclusions	No data was excluded from analyses.
Replication	All the experiments using cell lines and mice were repeat for three times. All attempts at replication were successful and all results were reproducible. Numbers of replications in different experiments can be found in the corresponding figure legends.
Randomization	In vitro samples and in vivo mouse models were divided into control (untreated or empty vector transfected), treatment groups or overexpression/knock-down. Patient samples were divided into treatment or non-treatment groups and study was conducted in a blinded manner. Immune different mice used in xenograft experiments were randomly assigned to experimental groups. For patient survival association with DUSP16 analysis, patients were divided into two groups based on DUSP16 protein expression levels followed by survival analysis using IBM SPSS Statistic 19.0.
Blinding	Immunohistological staining of patient samples was blinded, without information of their age, gender, HPV status or treatment status. The immunohistochemically stained cancer tissue sections were reviewed and scored by 1 experienced pathologist and 1 scientist blinded to the clinicopathological variables. No blinding of samples was performed in other experiments.

Reporting for specific materials, systems and methods

We require information from authors about some types of materials, experimental systems and methods used in many studies. Here, indicate whether each material, system or method listed is relevant to your study. If you are not sure if a list item applies to your research, read the appropriate section before selecting a response.

Materials & experimental systems

n/a	Involved in the study	
☐	☒	Antibodies
☐	☒	Eukaryotic cell lines
☒	☐	Palaeontology
☐	☒	Animals and other organisms
☐	☒	Human research participants
☒	☐	Clinical data

Methods

n/a	Involved in the study	
☒	☐	ChIP-seq
☐	☒	Flow cytometry
☒	☐	MRI-based neuroimaging

Antibodies

Antibodies used	Annexin V-FITC and 7-AAD (BioLegend) for apoptosis assay. β-actin (clone 13E5) Cell Signalling #4970, Bax Cell Signalling 2772, c-myc (Y69) Abcam#32072, Cleaved Caspase 3 (Asp175) Cell Signalling #9661, Cleaved Caspase 9 (Asp330) Cell Signalling #7237, Cytovhrome c Cell Signalling #4272, ERK (137F5) Cell Signalling #4695, JNK Cell Signalling #9252, MKP-7/DUSP16 (D5F4) Cell Signalling #5523, p38 Cell Signalling #9212, Phospho-ERK (Thr202/Tyr204) (197G2) Cell Signalling #4377, Phospho-JNK (Thr183/Tyr185) (98F2) Cell Signalling #4671, Phospho-p38 (Thr180/Tyr182) (3D7) Cell Signalling #9215, VDAC (D73D12) Cell Signalling #4661, ATF2 (20F1) Cell signaling #9226, Anti-rabbit HRP-conjudgated secondary GE Healthcare #NA934 for western analysis.
Validation	Examination of several cell lines and/or tissues of known expression levels allows accurate determination of species cross-reactivity and verifies specificity.<br>Treatment of cell lines with growth factors, chemical activators or inhibitors, which induce or inhibit target expression, verifies specificity. Phosphatase treatment confirms phospho-specificity.<br>The use of siRNA transfection or knockout cell lines verifies target specificity.<br>Side-by-side comparison of lots to ensures lot-to-lot consistency.<br>Optimal dilutions and buffers are predetermined, positive and negative cell extracts are specified, and detailed protocols are already optimized, saving valuable time and reagents.

Eukaryotic cell lines

Policy information about cell lines

Cell line source(s)	DLD-1 and HCT116 colorectal carcinoma cell lines , MDA-MB-231 breast cancer cell line, Ags gastric cancer cell line were from ATCC. HK-1 and C666-1 NPC cell lines were from Professor Chan Soh Ha (NUS Immunology Centre).
Authentication	no authentification

Mycoplasma contamination	Cells were tested negative for mycoplasma
Commonly misidentified lines (See ICLAC register)	No commonly misdidentified lines were used in this study.

Animals and other organisms

Policy information about studies involving animals; ARRIVE guidelines recommended for reporting animal research

Laboratory animals	NOD SCID gamma (NSG) mice (both male and female) at the age of 6 weeks were obtained from the Centre of Animal Resources (CARE)
Wild animals	No wild animals were used in this study.
Field-collected samples	No field-collected samples were used in this study.
Ethics oversight	Studies and procedures studies were conducted in accordance with the National University of Singapore (NUS) Institutional Animal Care and Use Committee (IACUC).

Note that full information on the approval of the study protocol must also be provided in the manuscript.

Human research participants

Policy information about studies involving human research participants

Population characteristics	*Describe the covariate-relevant population characteristics of the human research participants (e.g. age, gender, genotypic information, past and current diagnosis and treatment categories). If you filled out the behavioural & social sciences study design questions and have nothing to add here, write "See above."*
Recruitment	*Describe how participants were recruited. Outline any potential self-selection bias or other biases that may be present and how these are likely to impact results.*
Ethics oversight	*Identify the organization(s) that approved the study protocol.*

Note that full information on the approval of the study protocol must also be provided in the manuscript.

Flow Cytometry

Plots

Confirm that:

- [x] The axis labels state the marker and fluorochrome used (e.g. CD4-FITC).
- [x] The axis scales are clearly visible. Include numbers along axes only for bottom left plot of group (a 'group' is an analysis of identical markers).
- [x] All plots are contour plots with outliers or pseudocolor plots.
- [x] A numerical value for number of cells or percentage (with statistics) is provided.

Methodology

Sample preparation	NPC, CRC, gastric and breast cancer cells were grown in 6-well plates at a density of 0.25 x 106 cells/well and treated with drug over 24 and 48 hours. Adhering and detached cells were collected at the end of treatment and washed with 3% BSA in PBS. Cells were then suspended in 100uL 1x binding buffer and stained with 5uL of Annexin V-FITC and 5uL of 7-AAD (BioLegend) per sample for 15 minutes in the dark at room temperature. 400µl binding buffer was then added before analysis by flow cytometry.
Instrument	4 Laser BD LSRFortessa Special Order Research Product
Software	Flowjo was used to analyze staining of samples
Cell population abundance	Viable cells displayed in most cases >90% unstained/viable cells. Treatment-mediated apoptosis displayed reductions in percentage of viable cells and increase in percentage of cells in early apoptosis or late apoptosis/necrosis by the gating strategies stated below.
Gating strategy	Samples were gated as viable cells (unstained), early apoptosis (annexin V+/7AAD- cells), and necrotic or in late apoptosis (stained by both annexin V and 7AAD). The increase in apoptotic cells was also reflected by changes in the light scatter properties for the untreated and treated tubes (FSC-A vs SSC-A plots). During apoptosis, cell shrinkage occurs, which is associated with a decrease in forward scatter. The formation of apoptotic vesicles in the cells during apoptosis may also lead to an increased side scatter profile.

- [x] Tick this box to confirm that a figure exemplifying the gating strategy is provided in the Supplementary Information.
