# Supplementary figures and images for: DUSP16 promotes cancer chemoresistance through regulation of mitochondria-mediated cell death
Source: Nat Commun. 2021 Apr 16;12:2284. doi: 10.1038/s41467-021-22638-7 (PMC8052345; doi:10.1038/s41467-021-22638-7)

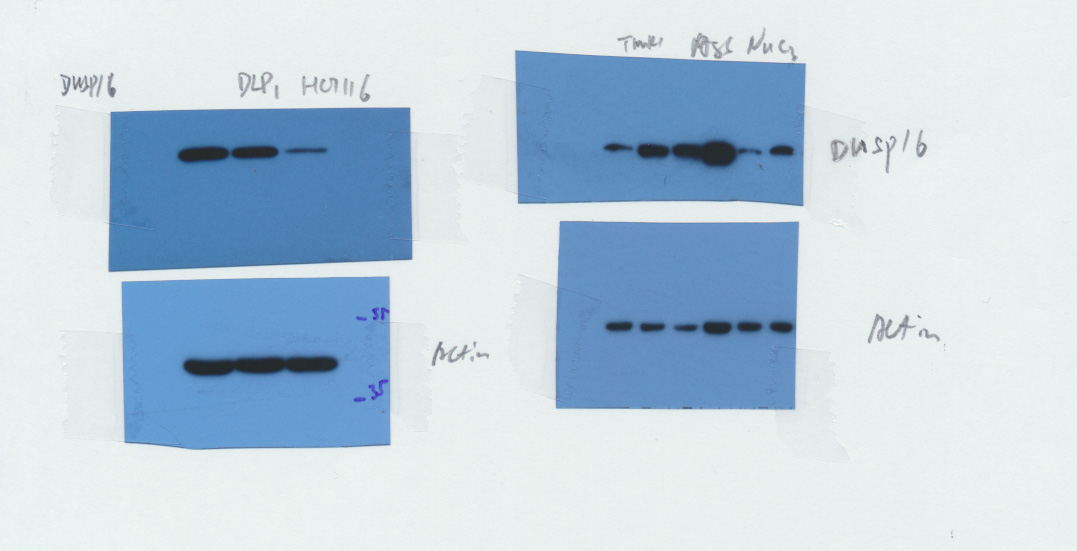

Supplement: Supplementary file 4 — Source Data [file 41467_2021_22638_MOESM4_ESM.zip › Source data files/Western blot images/Fig 1E-1F.jpg]

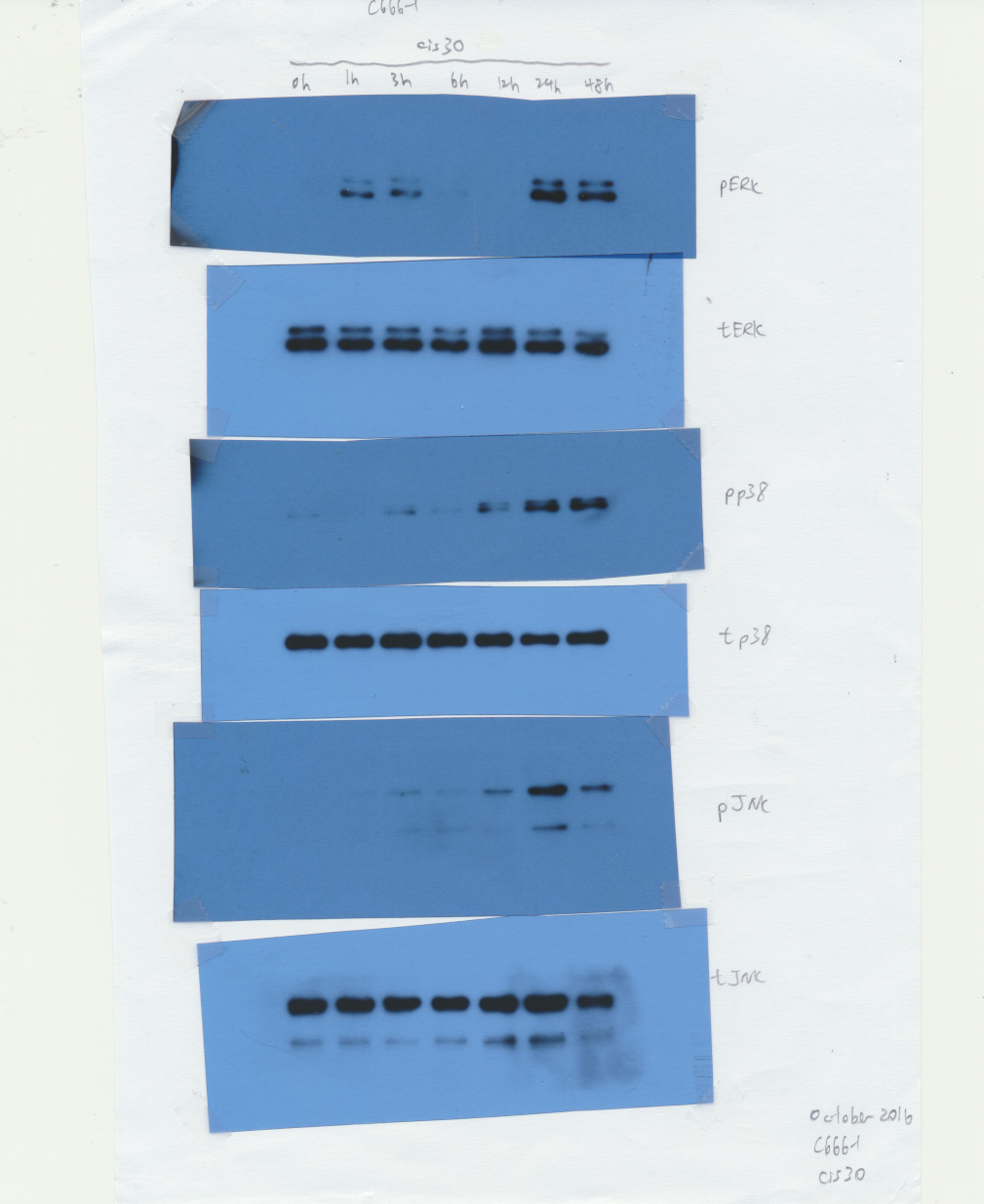

Supplement: Supplementary file 4 — Source Data [file 41467_2021_22638_MOESM4_ESM.zip › Source data files/Western blot images/Fig 1H.jpg]

2A

HK-1 DUSP16 clones

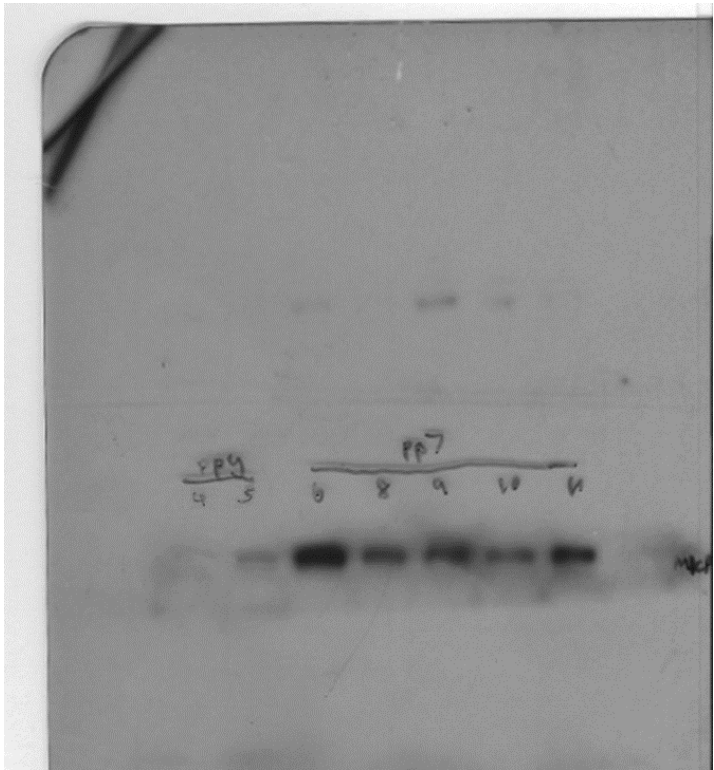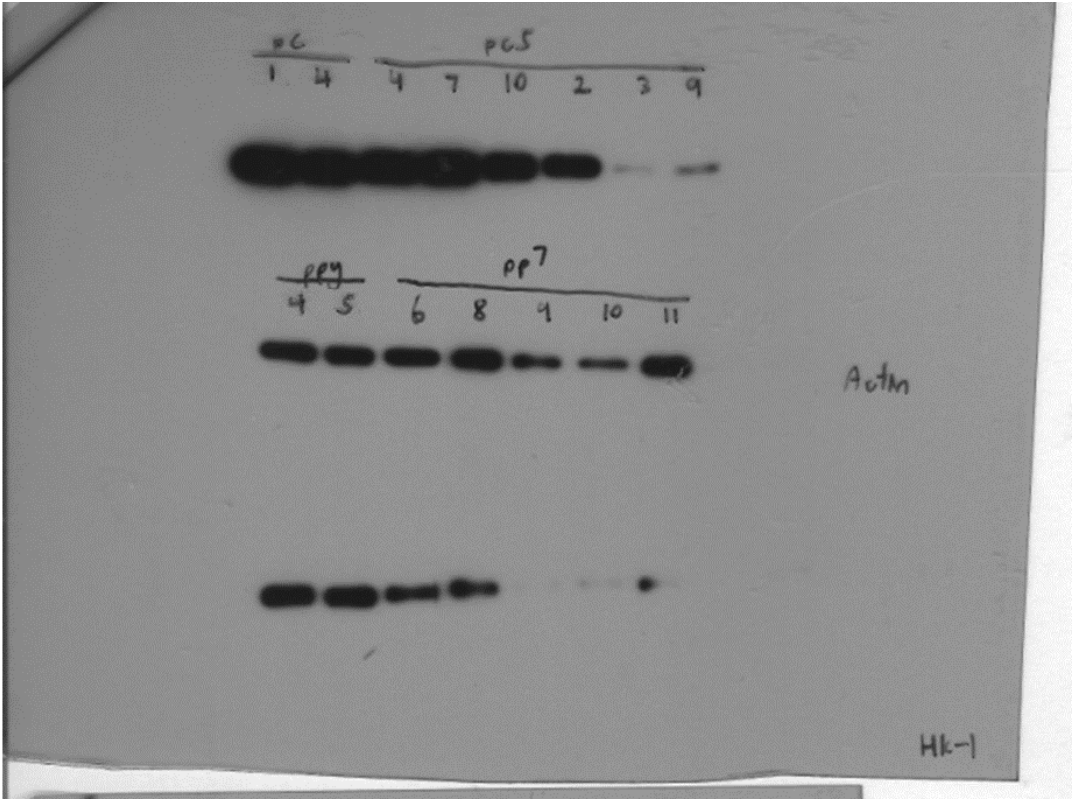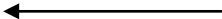

Supplement: Supplementary file 4 — Source Data [file 41467_2021_22638_MOESM4_ESM.zip › Source data files/Western blot images/Fig 2A-HK-1 DUSP16 OE clones.pdf]

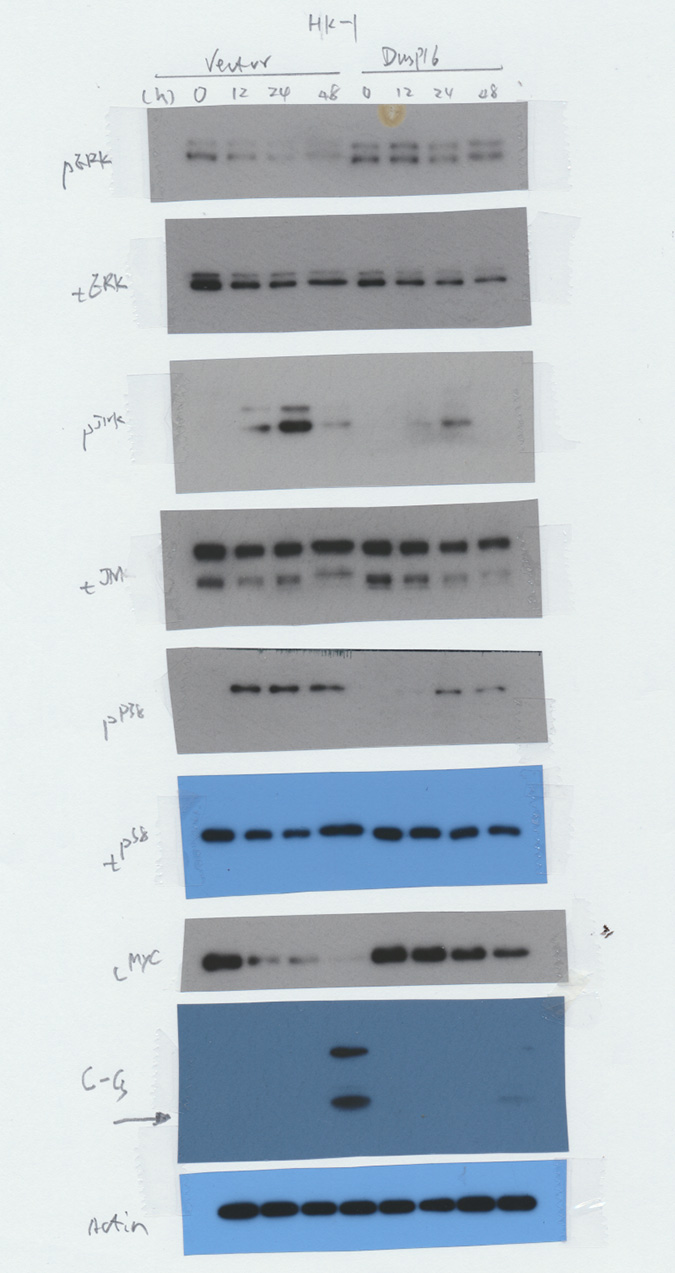

Supplement: Supplementary file 4 — Source Data [file 41467_2021_22638_MOESM4_ESM.zip › Source data files/Western blot images/Fig 4A-HK1.jpg]

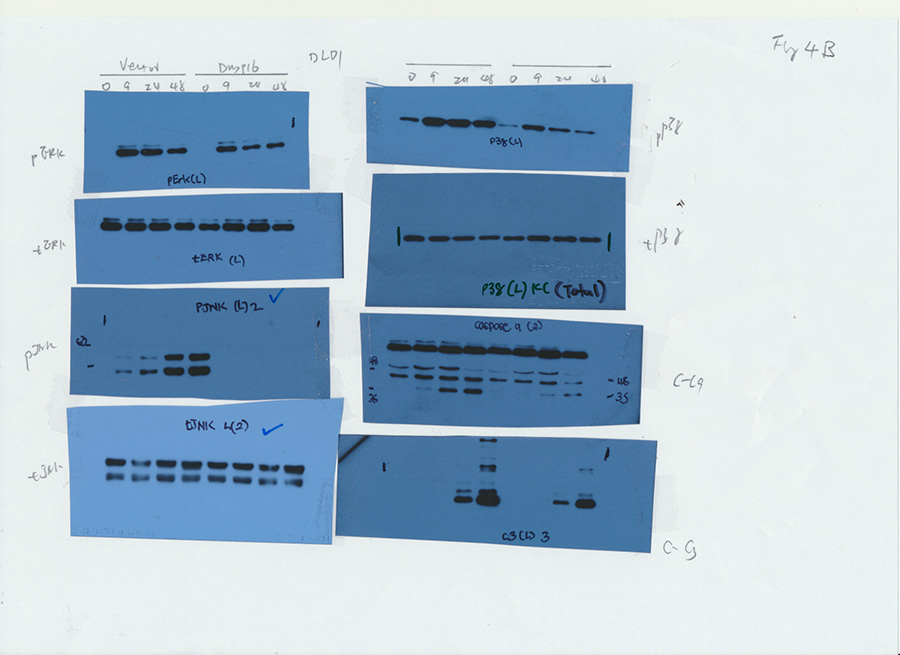

Supplement: Supplementary file 4 — Source Data [file 41467_2021_22638_MOESM4_ESM.zip › Source data files/Western blot images/Fig 4B.jpg]

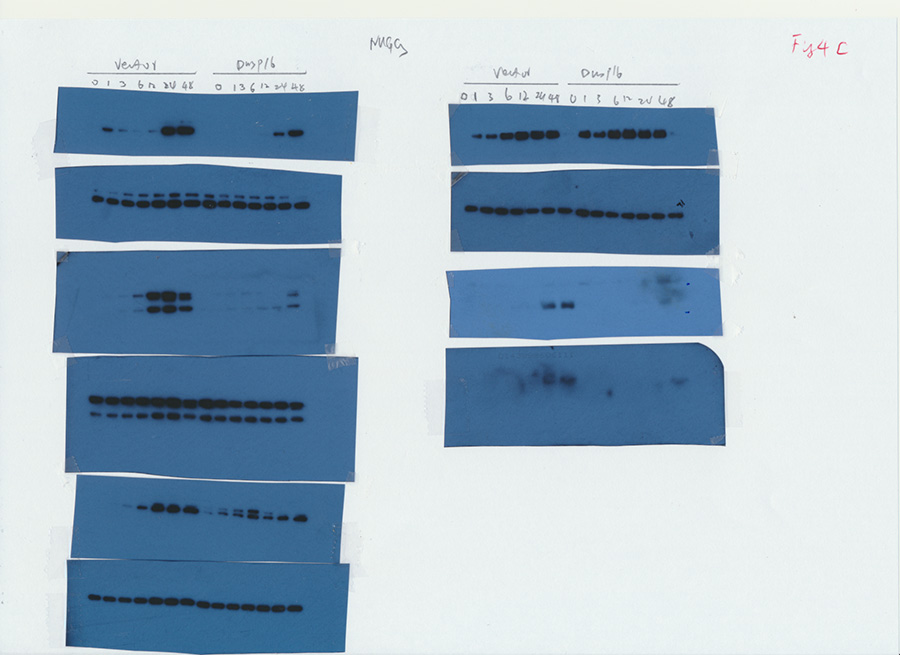

Supplement: Supplementary file 4 — Source Data [file 41467_2021_22638_MOESM4_ESM.zip › Source data files/Western blot images/Fig 4C-NugC3.jpg]

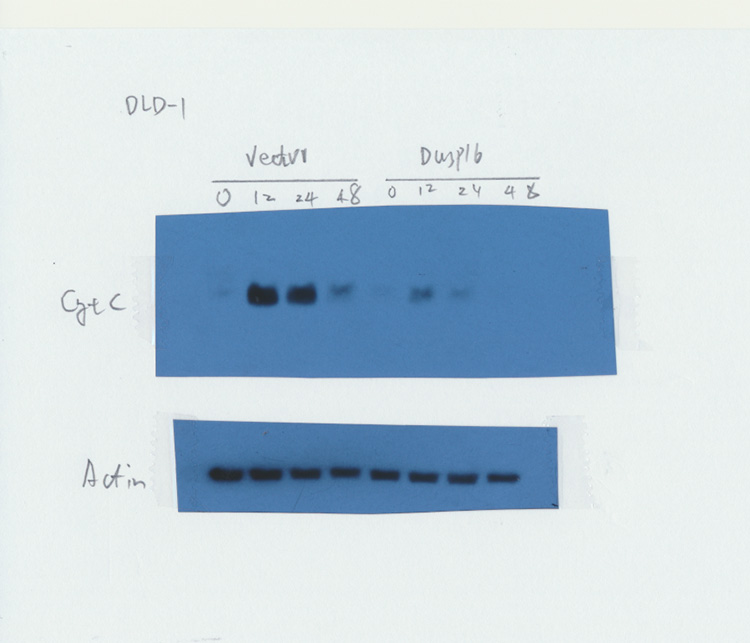

Supplement: Supplementary file 4 — Source Data [file 41467_2021_22638_MOESM4_ESM.zip › Source data files/Western blot images/Fig 4D-B.jpg]

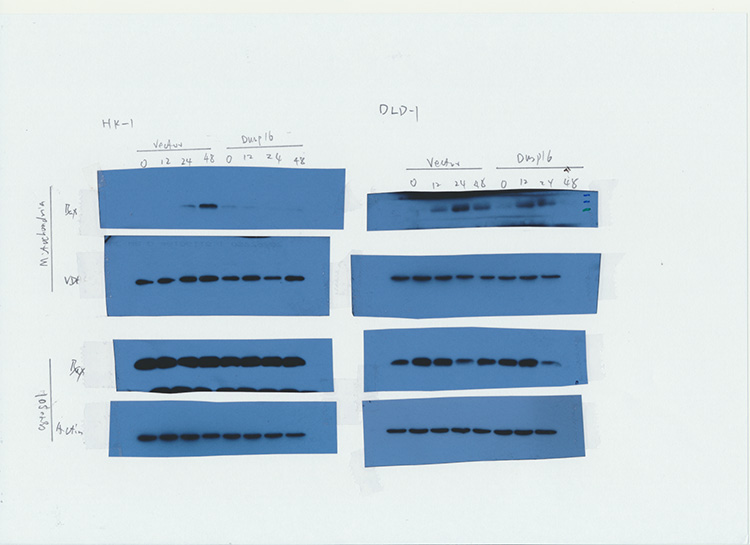

Supplement: Supplementary file 4 — Source Data [file 41467_2021_22638_MOESM4_ESM.zip › Source data files/Western blot images/Fig 4F.jpg]

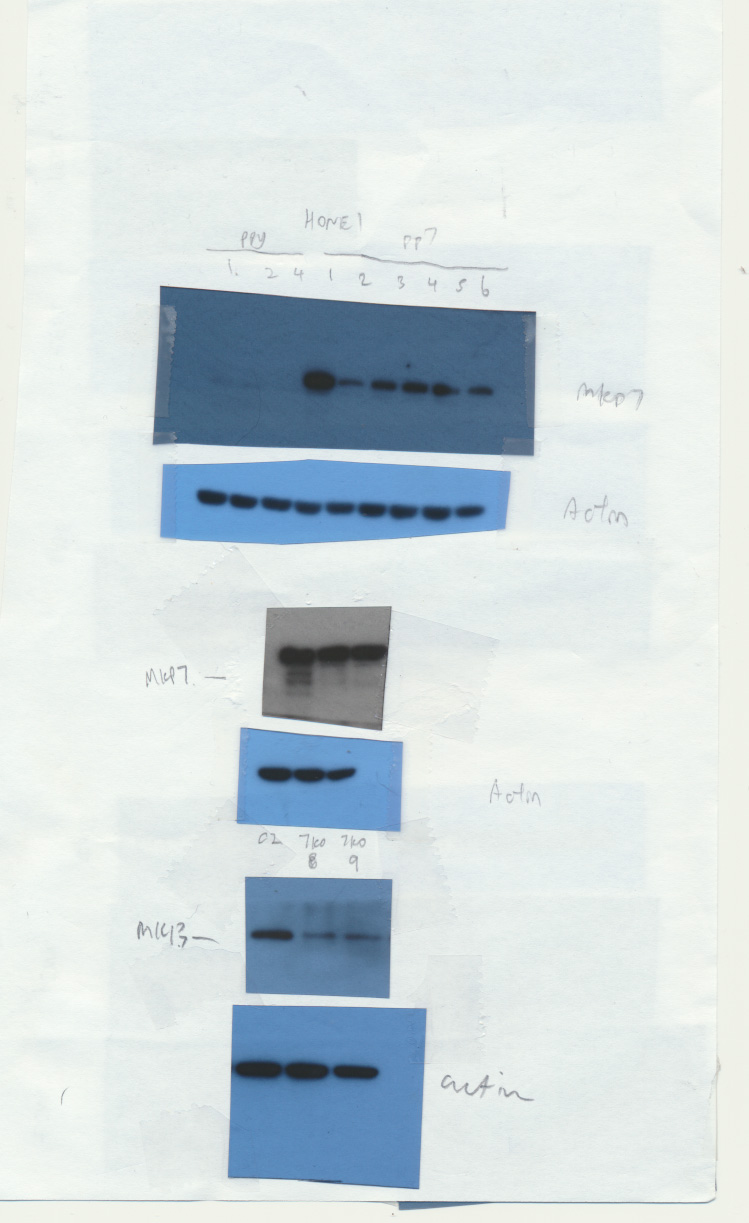

Supplement: Supplementary file 4 — Source Data [file 41467_2021_22638_MOESM4_ESM.zip › Source data files/Western blot images/Fig 6A.jpg]

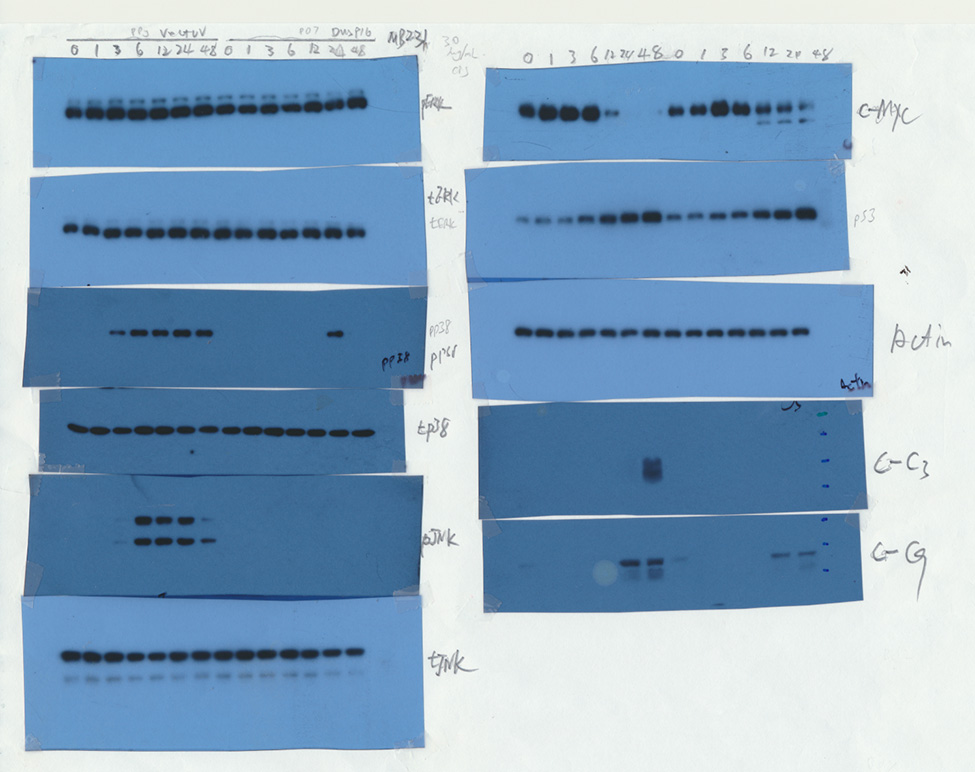

Supplement: Supplementary file 4 — Source Data [file 41467_2021_22638_MOESM4_ESM.zip › Source data files/Western blot images/Fig S2-D.jpg]

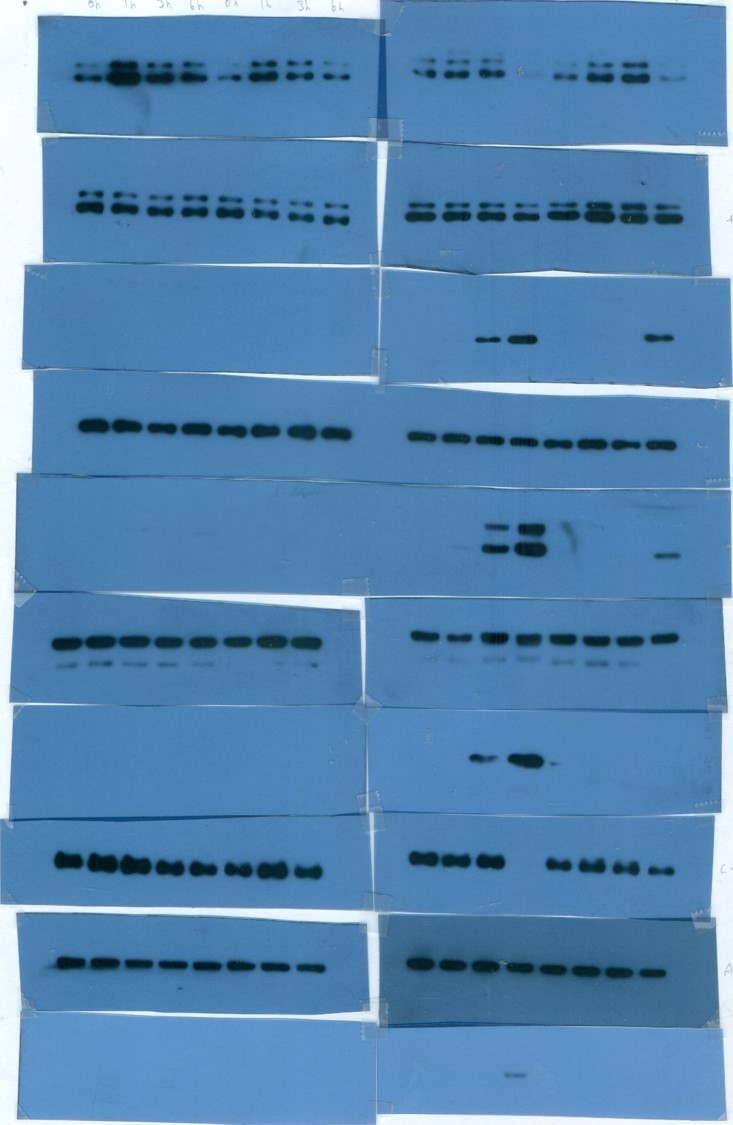

Supplement: Supplementary file 4 — Source Data [file 41467_2021_22638_MOESM4_ESM.zip › Source data files/Western blot images/Fig S3C.jpg]

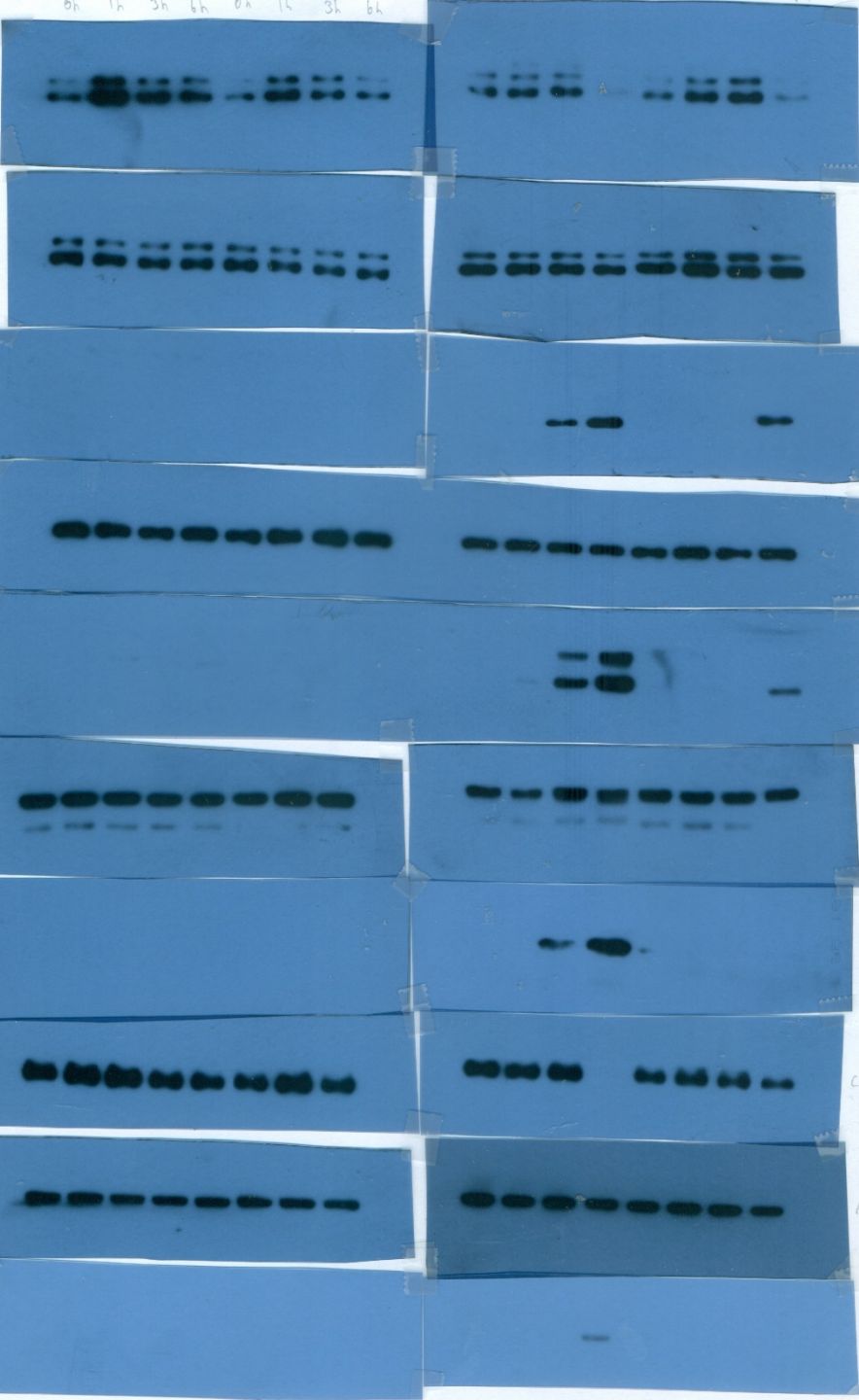

Caspase 9

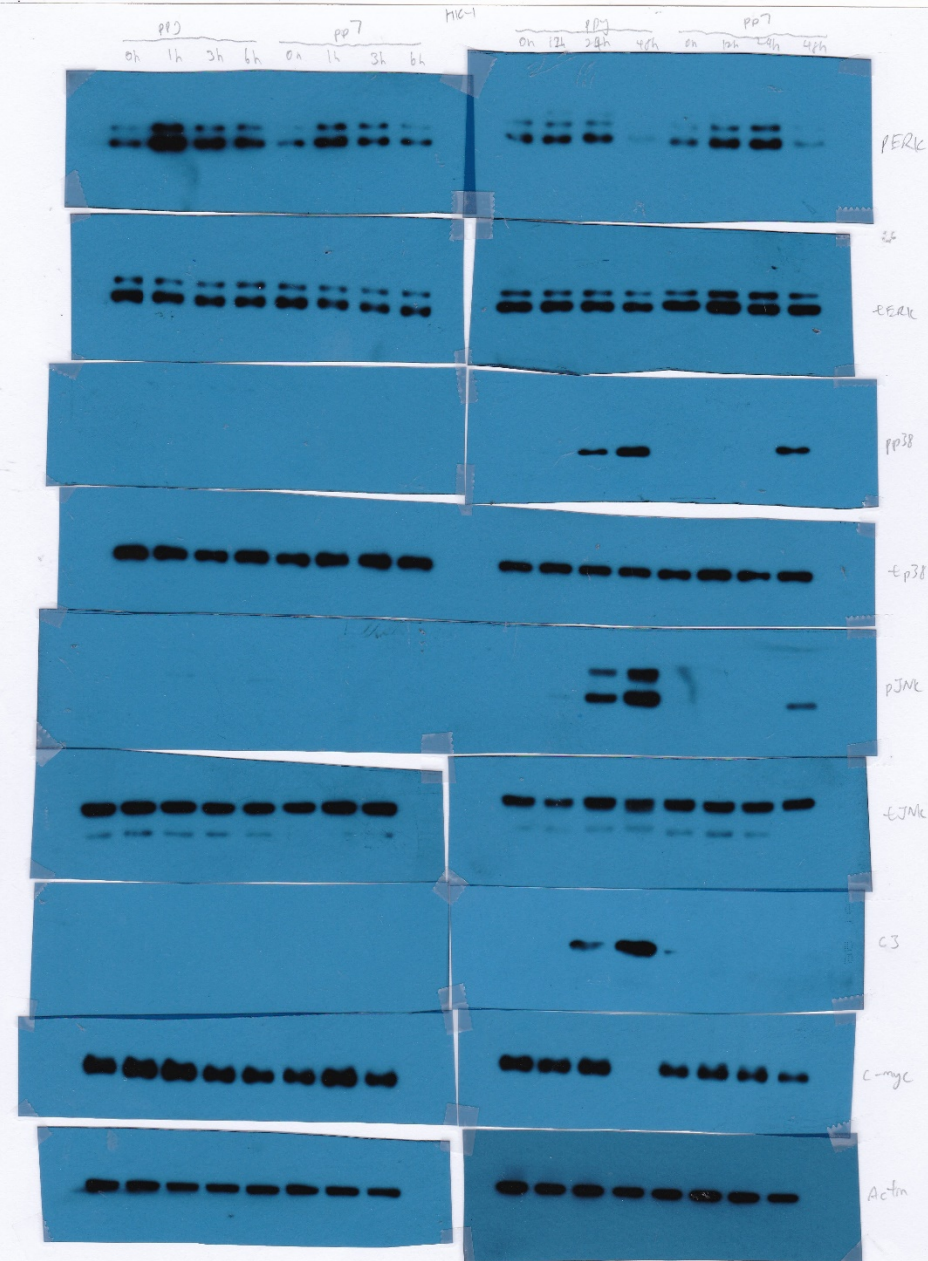

HK-1  
Carboplatin (200uM)

S3C

Supplement: Supplementary file 4 — Source Data [file 41467_2021_22638_MOESM4_ESM.zip › Source data files/Western blot images/Fig S3C.pdf]

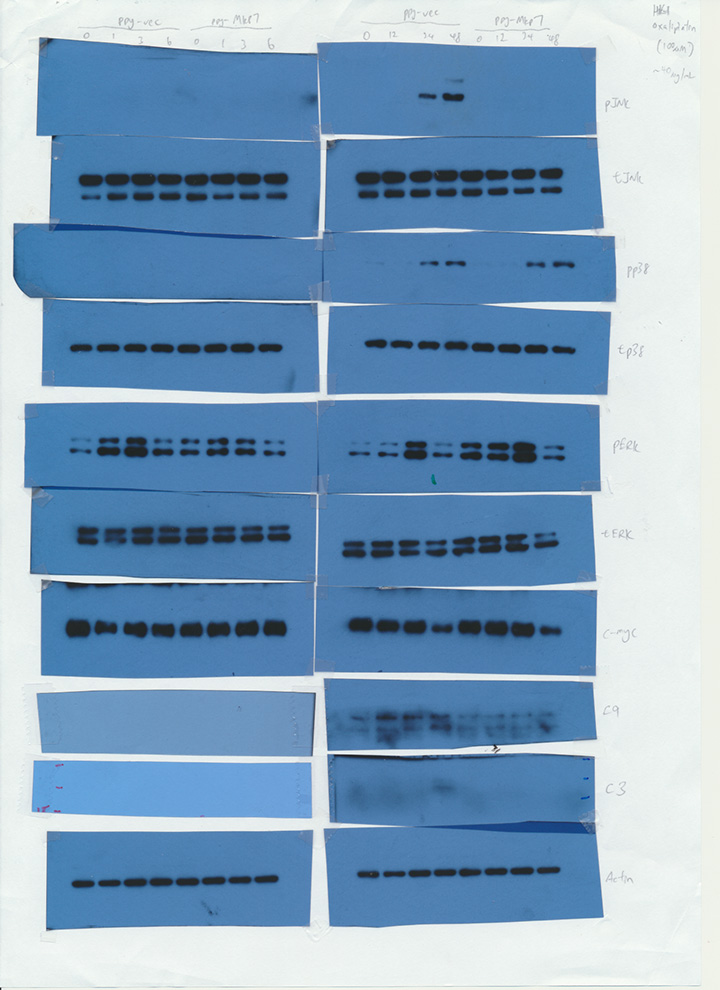

Supplement: Supplementary file 4 — Source Data [file 41467_2021_22638_MOESM4_ESM.zip › Source data files/Western blot images/Fig S3D-HK-1 to Oxaliplatin.jpg]

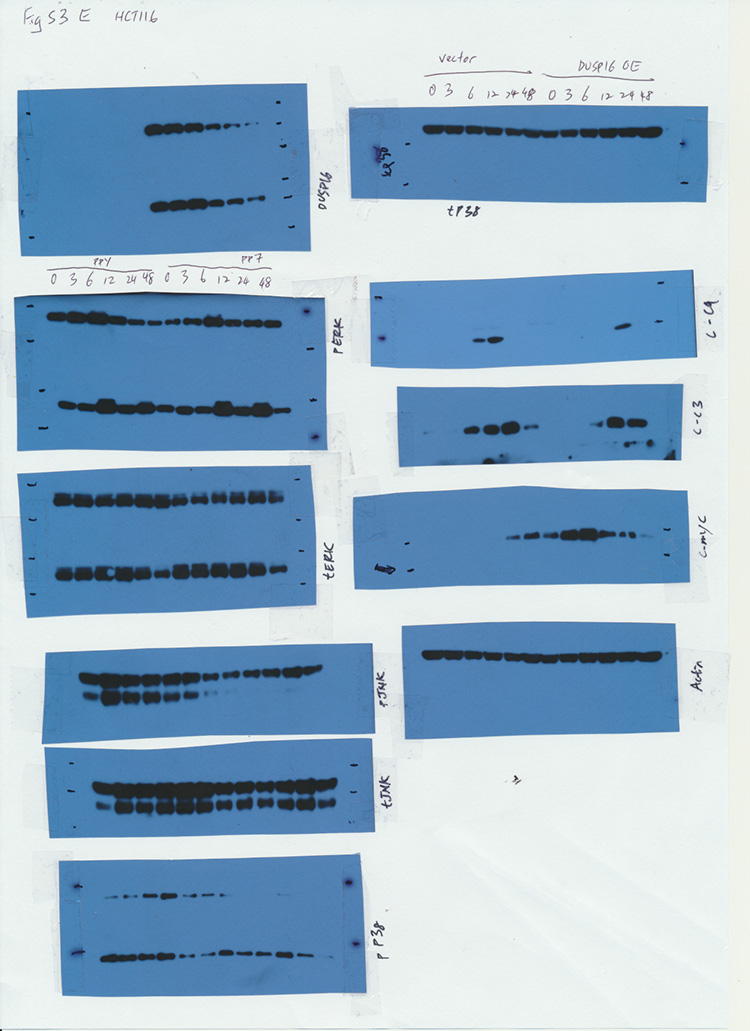

Supplement: Supplementary file 4 — Source Data [file 41467_2021_22638_MOESM4_ESM.zip › Source data files/Western blot images/Fig S3E-HCT116.jpg]

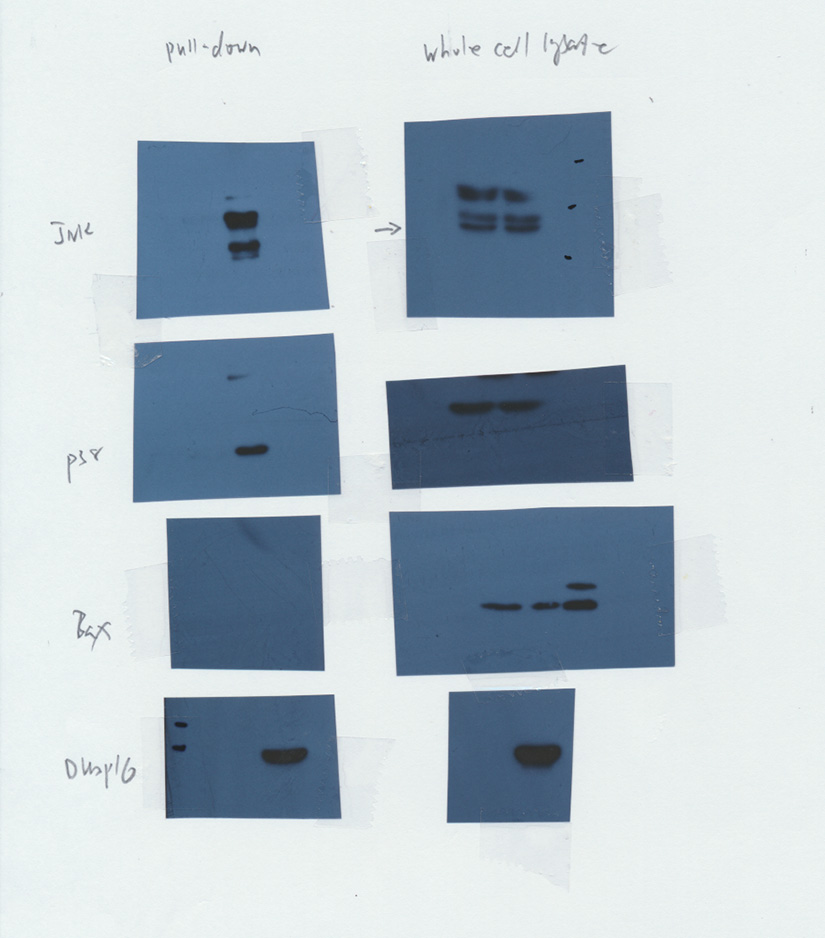

Supplement: Supplementary file 4 — Source Data [file 41467_2021_22638_MOESM4_ESM.zip › Source data files/Western blot images/Fig S5A IP.jpg]

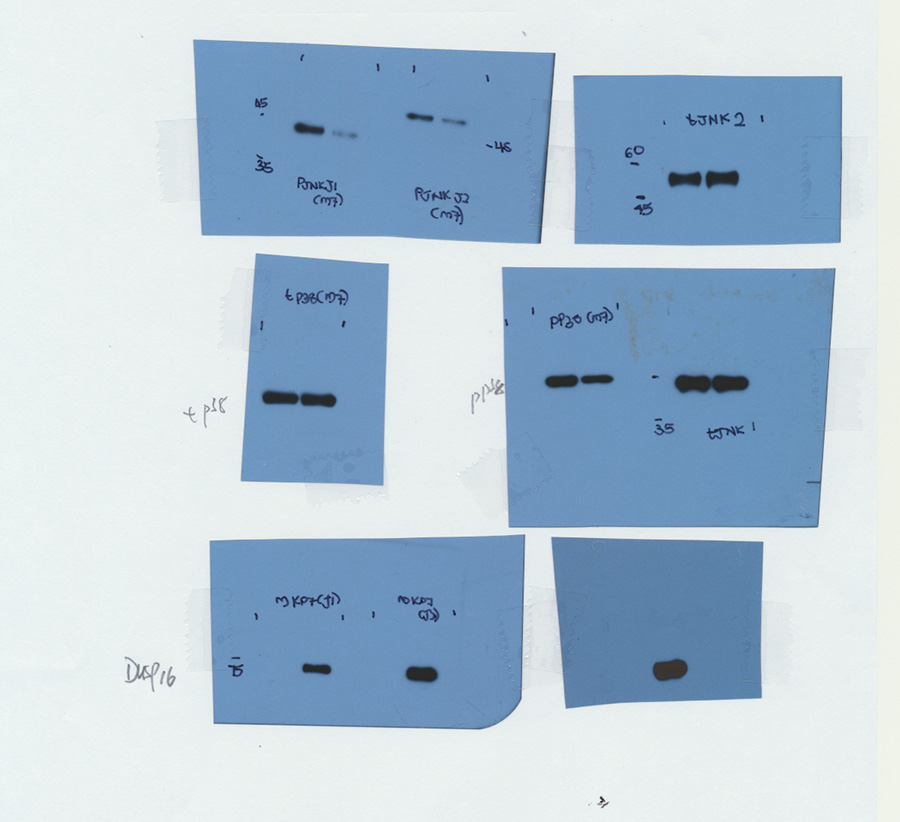

Supplement: Supplementary file 4 — Source Data [file 41467_2021_22638_MOESM4_ESM.zip › Source data files/Western blot images/Fig S5B.jpg]

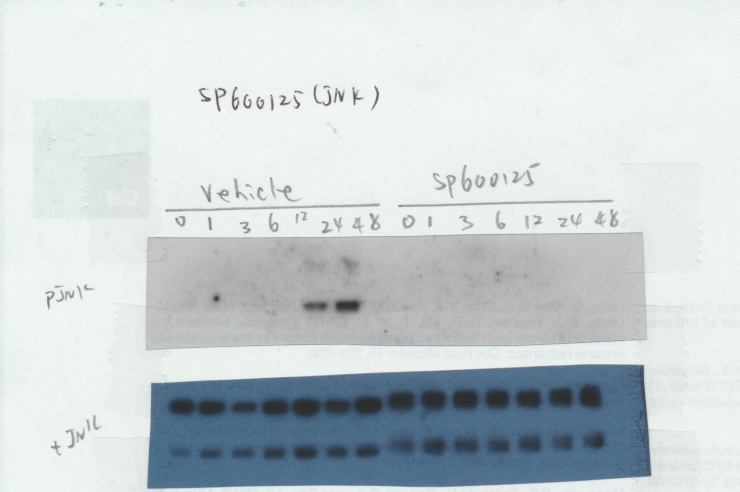

Supplement: Supplementary file 4 — Source Data [file 41467_2021_22638_MOESM4_ESM.zip › Source data files/Western blot images/Fig S5C-JNK inhibition.jpg]

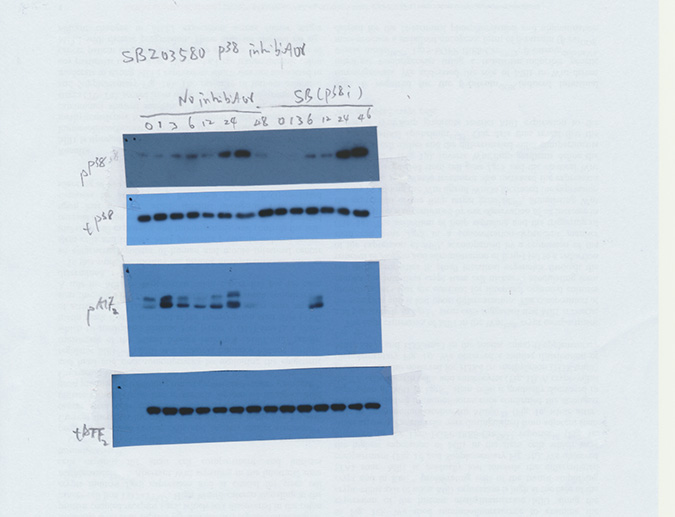

Supplement: Supplementary file 4 — Source Data [file 41467_2021_22638_MOESM4_ESM.zip › Source data files/Western blot images/Fig S5D-p38 inhibition.jpg]

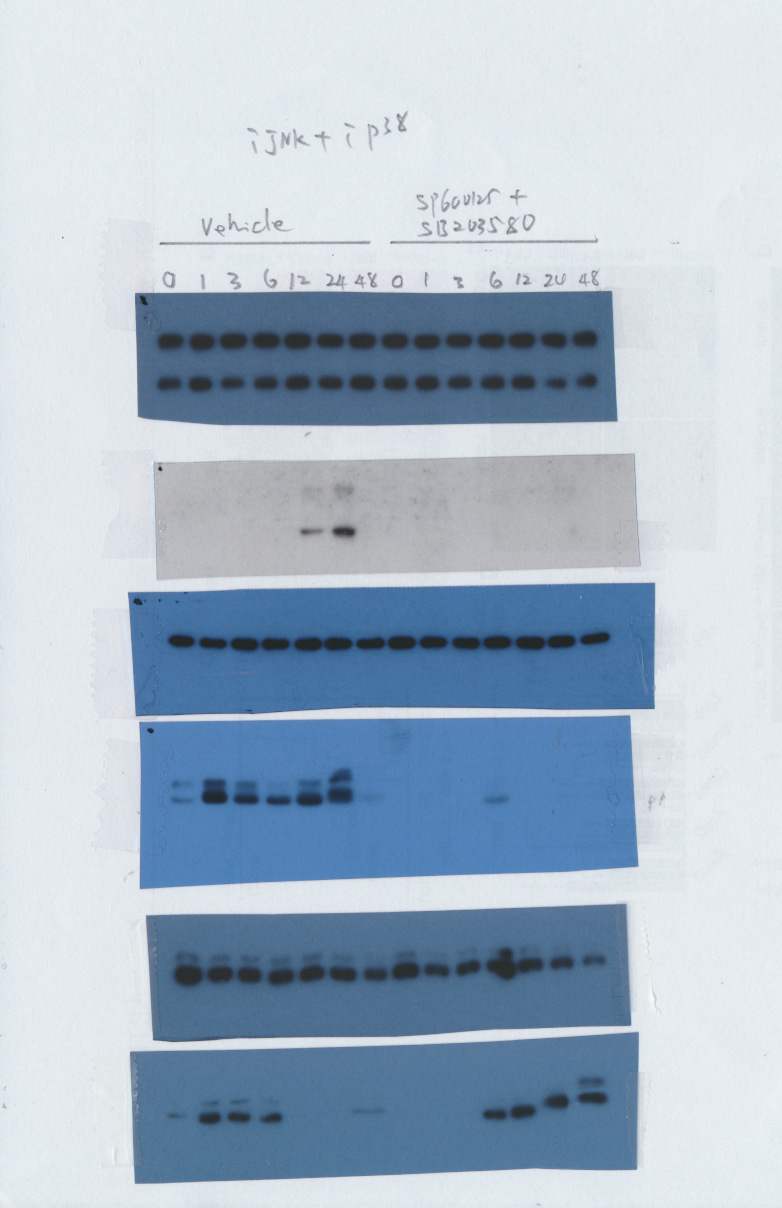

Supplement: Supplementary file 4 — Source Data [file 41467_2021_22638_MOESM4_ESM.zip › Source data files/Western blot images/Fig S5E-JNK and p38 double inhibition.jpg]

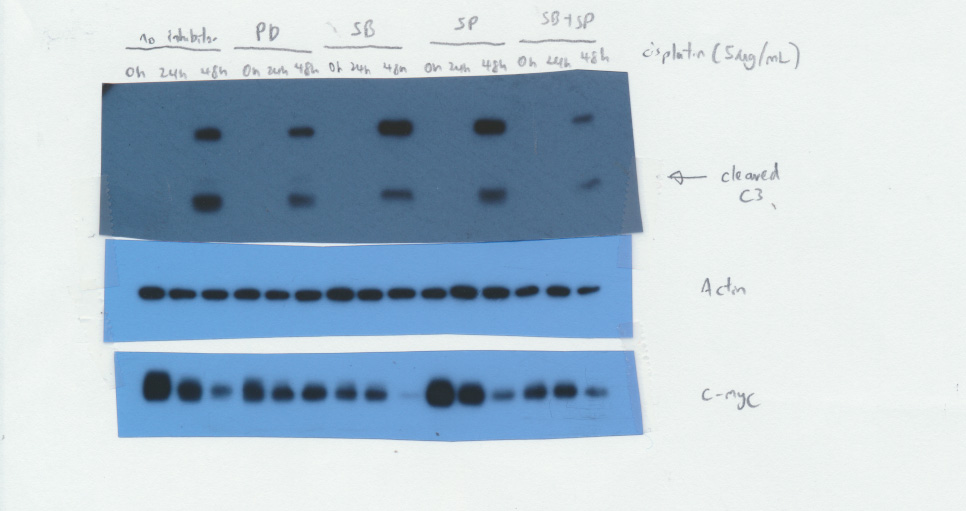

Supplement: Supplementary file 4 — Source Data [file 41467_2021_22638_MOESM4_ESM.zip › Source data files/Western blot images/Fig S5G-C3 and C-Myc.jpg]

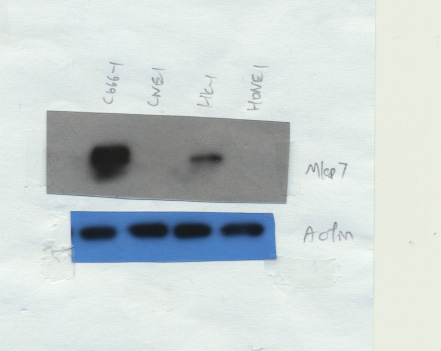

Supplement: Supplementary file 4 — Source Data [file 41467_2021_22638_MOESM4_ESM.zip › Source data files/Western blot images/Fig1B.jpg]

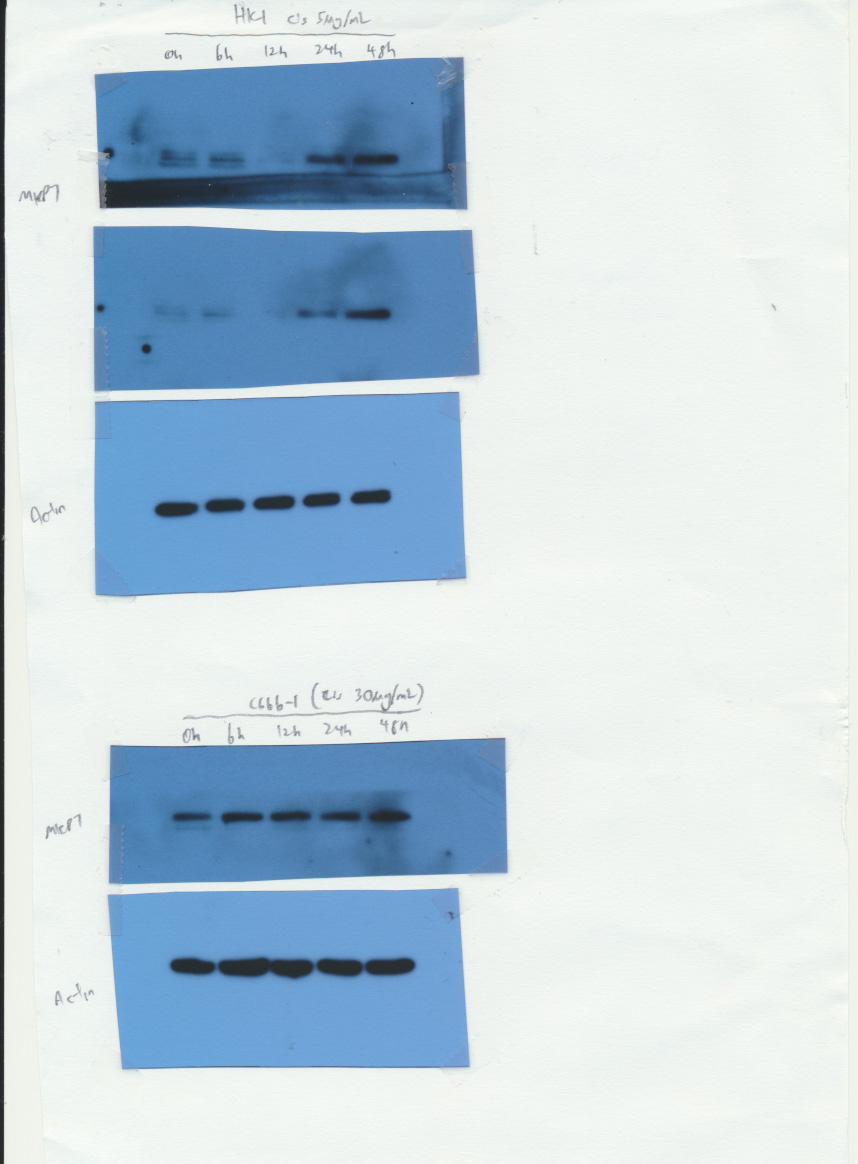

Supplement: Supplementary file 4 — Source Data [file 41467_2021_22638_MOESM4_ESM.zip › Source data files/Western blot images/Fig1C.jpg]

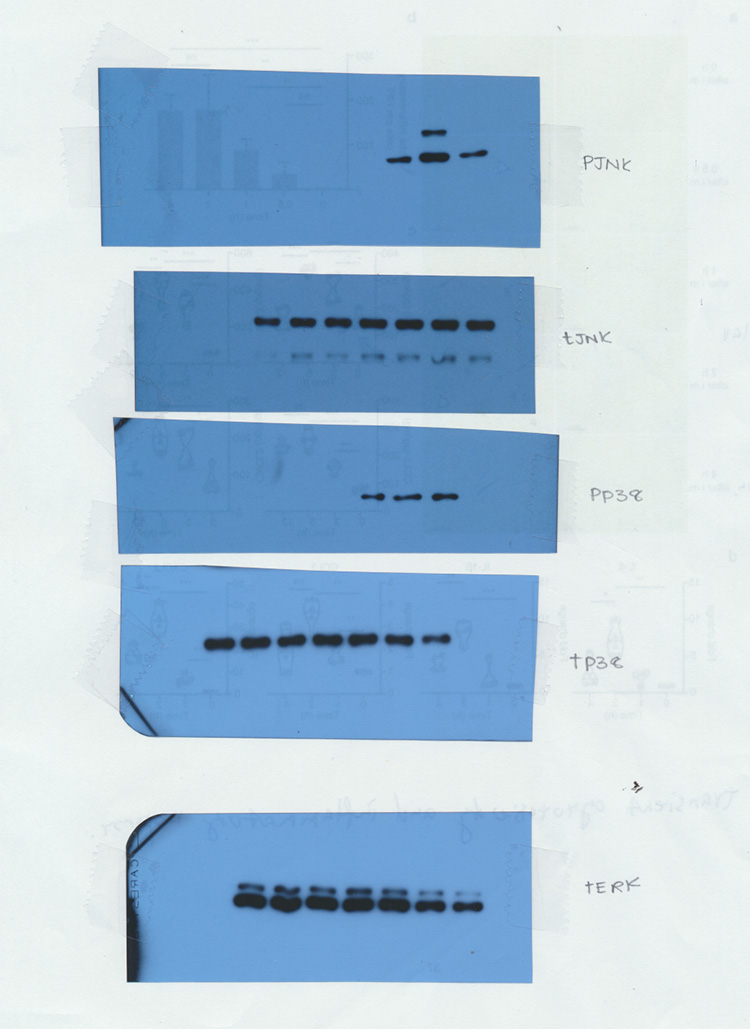

Supplement: Supplementary file 4 — Source Data [file 41467_2021_22638_MOESM4_ESM.zip › Source data files/Western blot images/Fig1G.jpg]

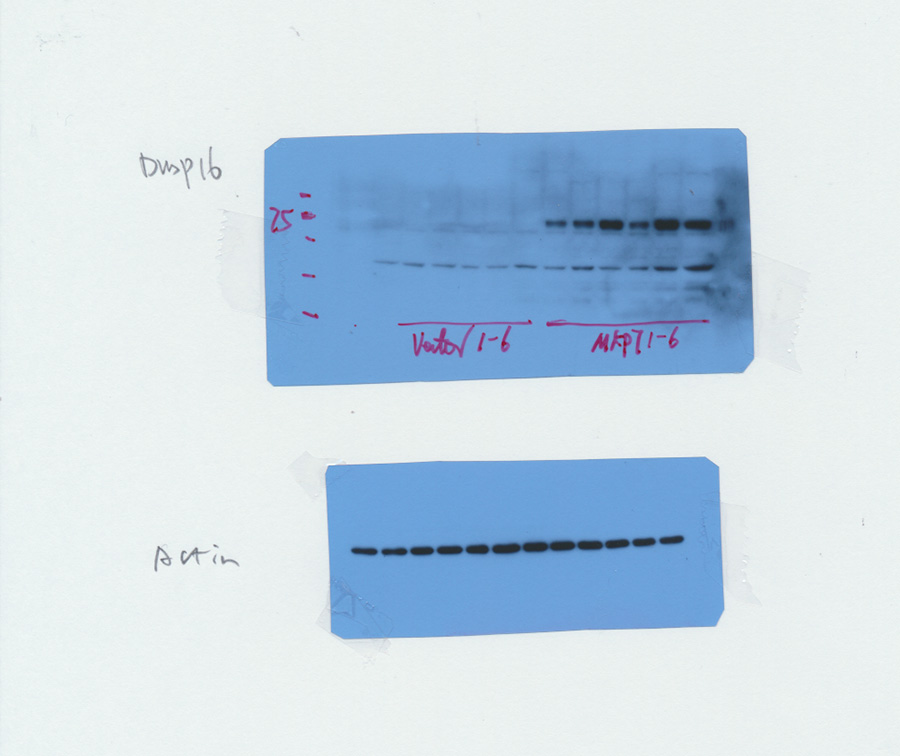

Supplement: Supplementary file 4 — Source Data [file 41467_2021_22638_MOESM4_ESM.zip › Source data files/Western blot images/Fig2D DLD1.jpg]

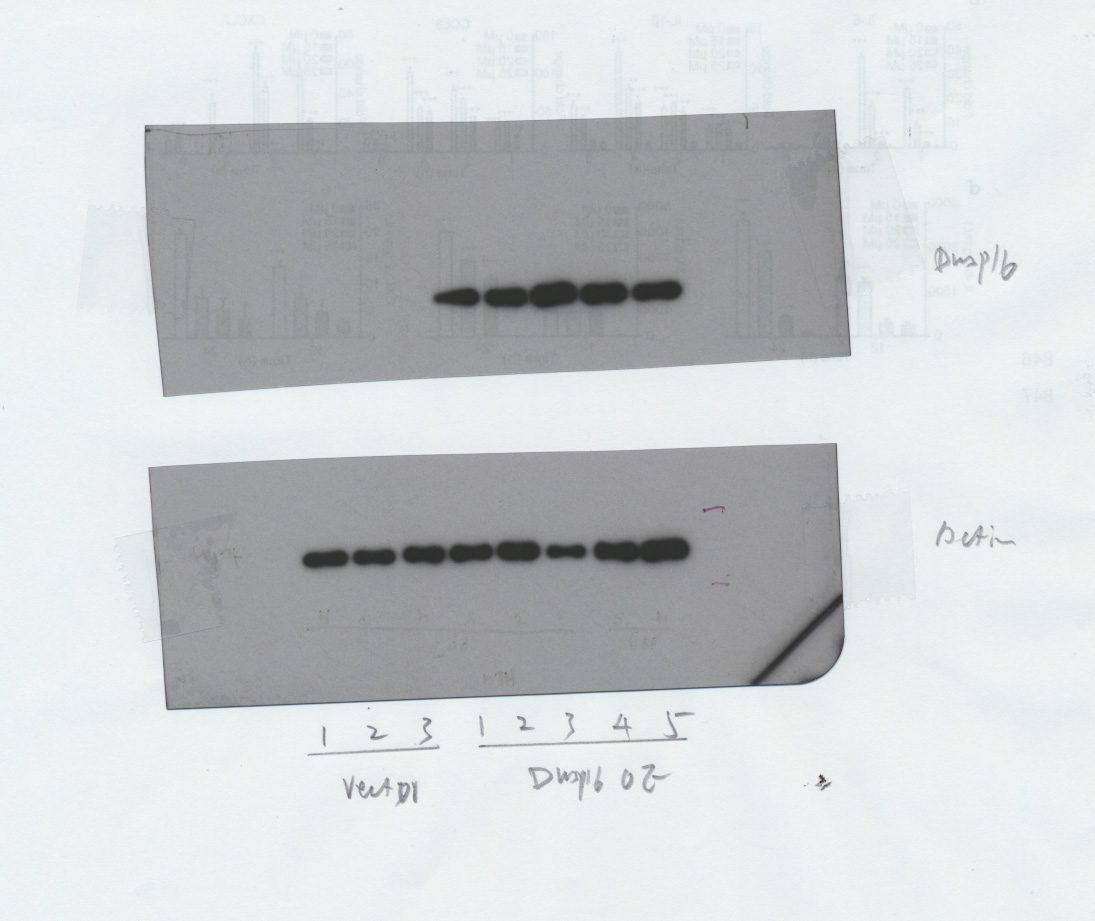

Supplement: Supplementary file 4 — Source Data [file 41467_2021_22638_MOESM4_ESM.zip › Source data files/Western blot images/Fig2G.jpg]

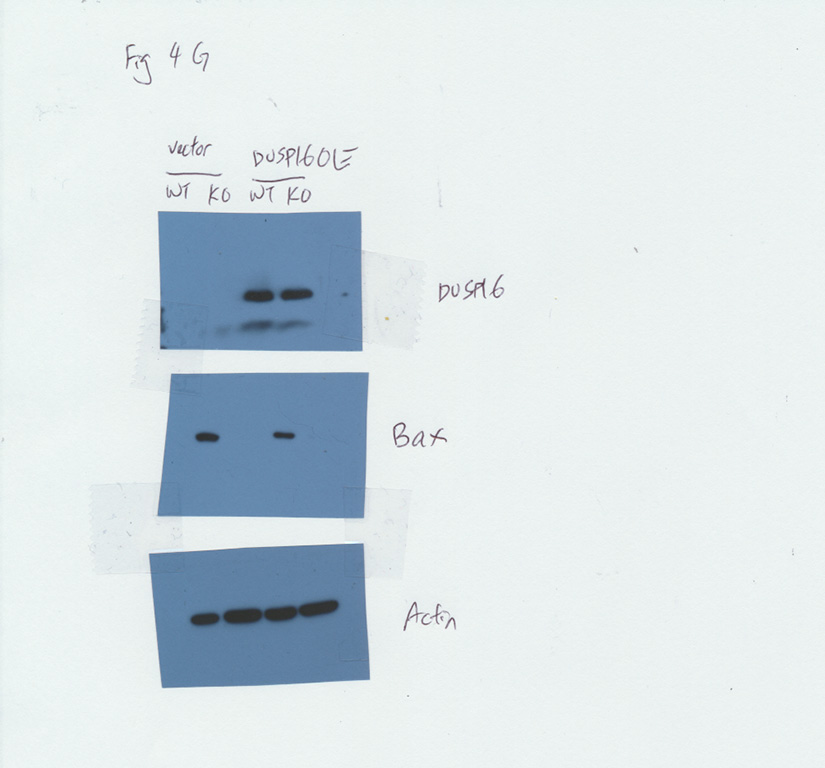

Supplement: Supplementary file 4 — Source Data [file 41467_2021_22638_MOESM4_ESM.zip › Source data files/Western blot images/Fig4G.jpg]

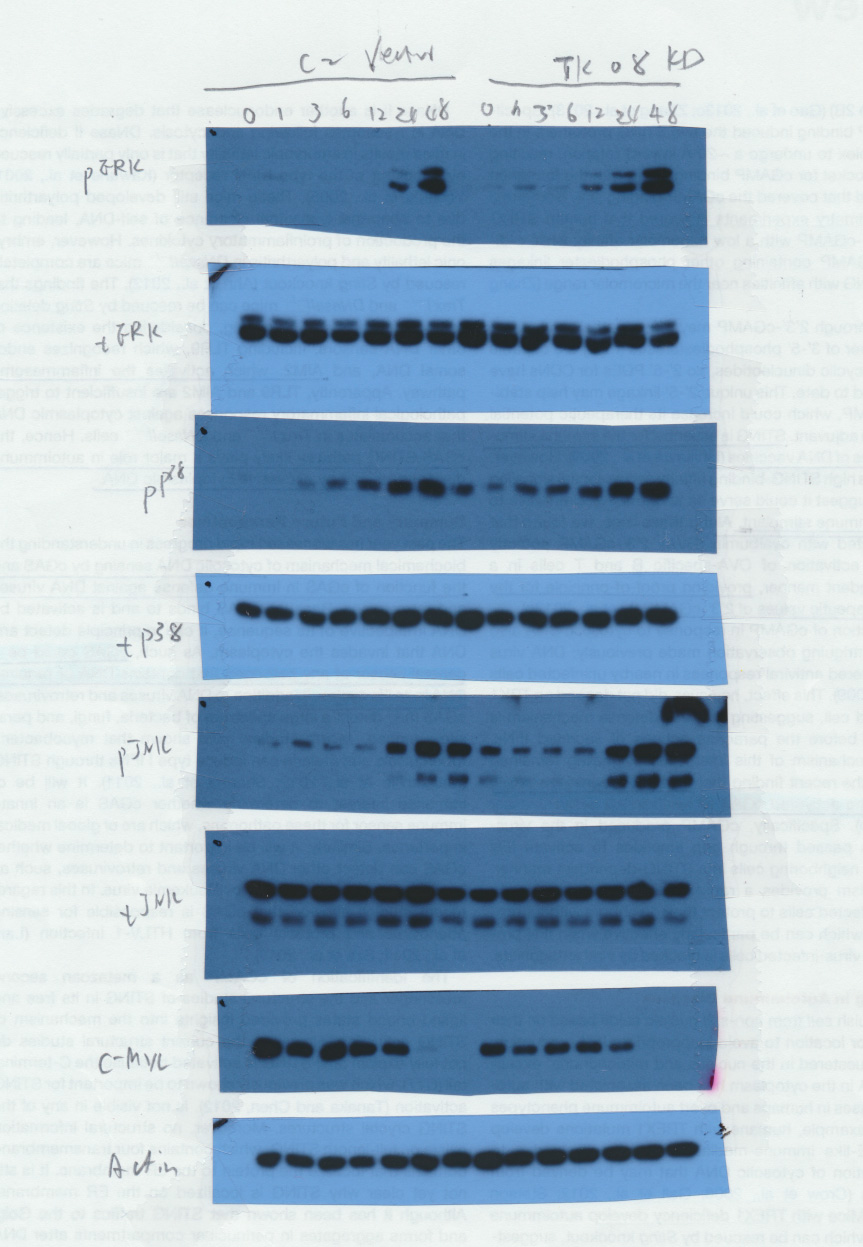

Supplement: Supplementary file 4 — Source Data [file 41467_2021_22638_MOESM4_ESM.zip › Source data files/Western blot images/Fig6D.jpg]

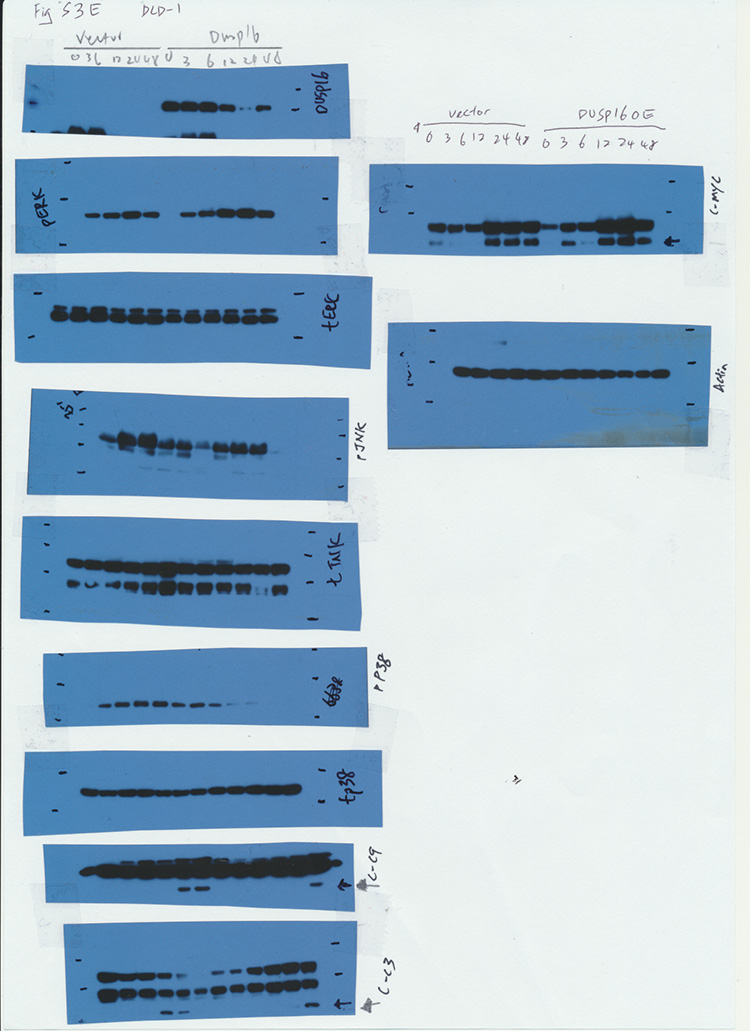

Supplement: Supplementary file 4 — Source Data [file 41467_2021_22638_MOESM4_ESM.zip › Source data files/Western blot images/FigS3E.jpg]

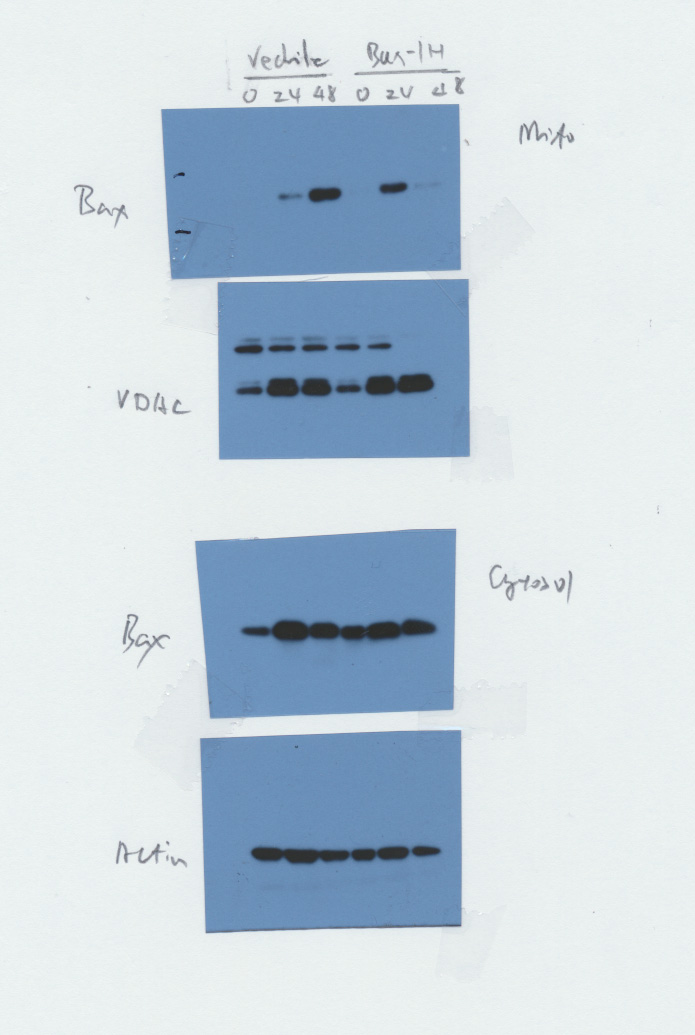

Supplement: Supplementary file 4 — Source Data [file 41467_2021_22638_MOESM4_ESM.zip › Source data files/Western blot images/FigS4 Bax inhibitor.jpg]

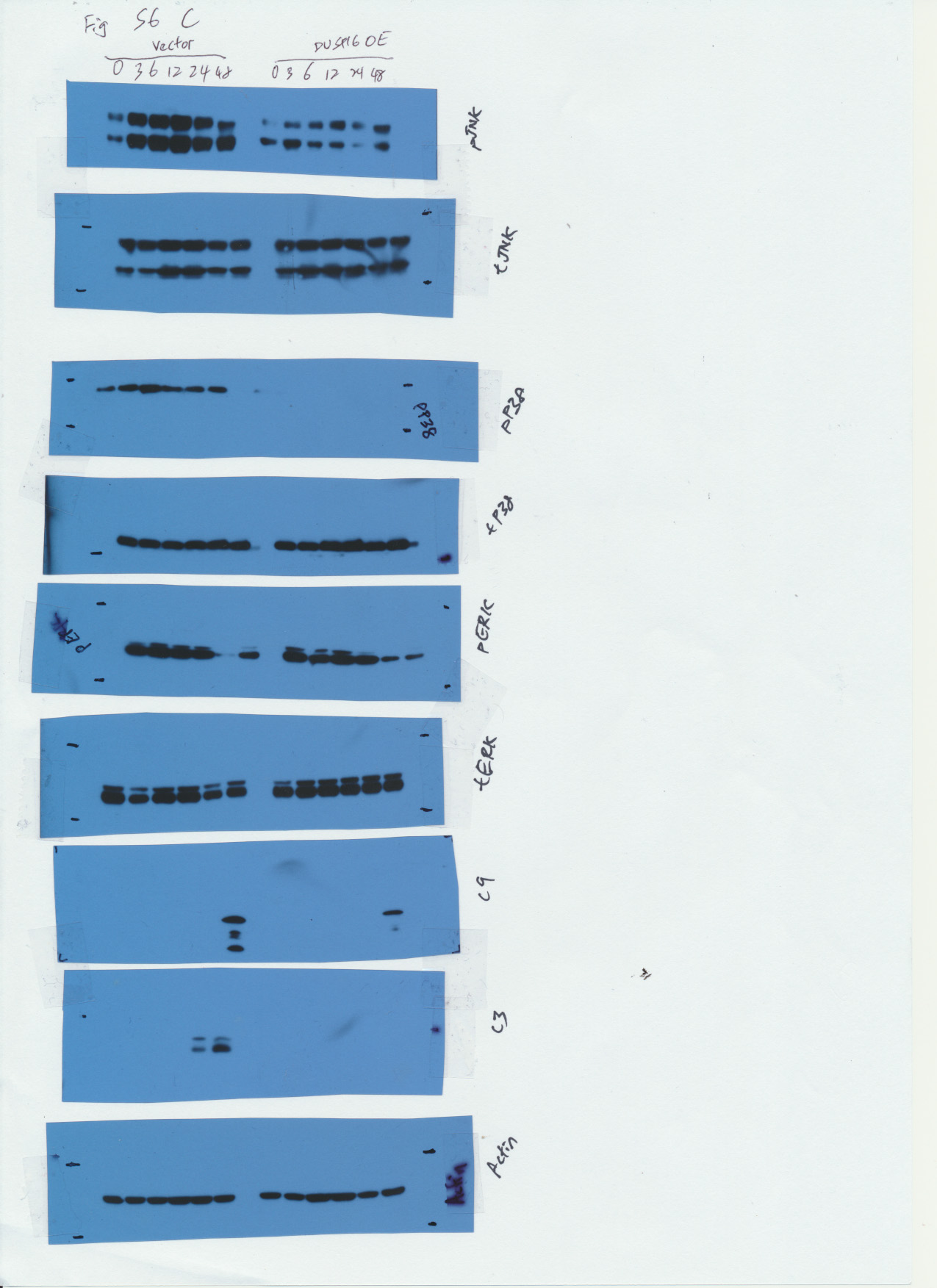

Supplement: Supplementary file 4 — Source Data [file 41467_2021_22638_MOESM4_ESM.zip › Source data files/Western blot images/FigS6C.jpg]

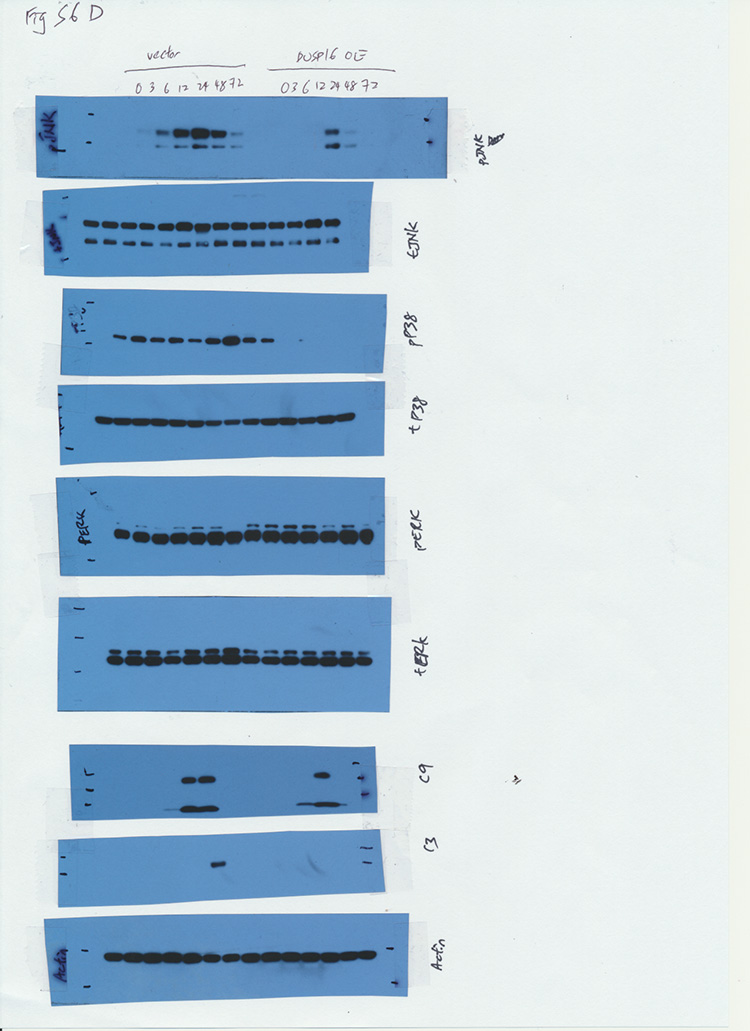

Supplement: Supplementary file 4 — Source Data [file 41467_2021_22638_MOESM4_ESM.zip › Source data files/Western blot images/FigS6D.jpg]
